# SynCAM1 deficiency in the hippocampal parvalbumin interneurons contributes to sevoflurane‐induced cognitive impairment in neonatal rats

**DOI:** 10.1111/cns.14554

**Published:** 2023-12-17

**Authors:** Ming‐ming Zhao, Han‐wen Gu, Wei‐tong Pan, Pan‐miao Liu, Ting‐ting Zhu, Hui‐jie Shang, Min Jia, Jian‐jun Yang

**Affiliations:** ^1^ Department of Anesthesiology, Pain and Perioperative Medicine The First Affiliated Hospital of Zhengzhou University Zhengzhou China; ^2^ Neuroscience Research Institute, Zhengzhou University Academy of Medical Sciences Zhengzhou University Zhengzhou China

**Keywords:** cognitive impairments, hippocampus, PV interneurons, sevoflurane, SynCAM1

## Abstract

**Aims:**

Sevoflurane is widely used for general anesthesia in children. Previous studies reported that multiple neonatal exposures to sevoflurane can induce long‐term cognitive impairment in adolescent rats, but the underlying mechanisms were not defined.

**Methods:**

Postnatal day 6 (P6) to P8 rat pups were exposed to 30% oxygen with or without 3% sevoflurane balanced with air. The Y maze test (YMT) and Morris water maze (MWM) tests were performed in some cohorts from age P35 to assess cognitive functions, and their brain samples were harvested at age P14, 21, 28, 35, and 42 for measurements of various molecular entities and in vivo electrophysiology experiments at age P35.

**Results:**

Sevoflurane exposure resulted in cognitive impairment that was associated with decreased synCAM1 expression in parvalbumin (PV) interneurons, a reduction of PV phenotype, disturbed gamma oscillations, and dendritic spine loss in the hippocampal CA3 region. Enriched environment (EE) increased synCAM1 expression in the PV interneurons and attenuated sevoflurane‐induced cognitive impairment. The synCAM1 overexpression by the adeno‐associated virus vector in the hippocampal CA3 region restored sevoflurane‐induced cognitive impairment, PV phenotype loss, gamma oscillations decrease, and dendritic spine loss.

**Conclusion:**

Our data suggested that neonatal sevoflurane exposure results in cognitive impairment through decreased synCAM1 expression in PV interneurons in the hippocampus.

## Graphical abstract

Neonatal exposure to repeated sevoflurane anesthesia resulted in cognitive impairment associated with decreased synCAM1 expression in the hippocampal PV interneurons, reduced PV interneurons phenotype, and disturbed gamma oscillations. Moreover, overexpression of synCAM1 in the hippocampal PV interneurons improved cognitive function by restoring PV expression and gamma oscillations after repeated sevoflurane exposure.

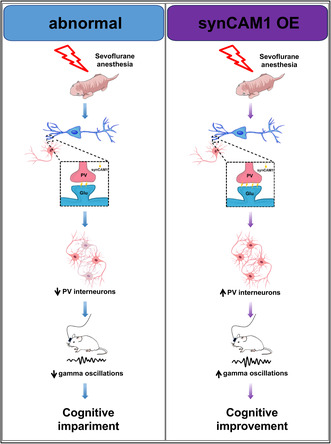

## 1 INTRODUCTION

Millions of children receive anesthesia for surgical and diagnostic procedures each year.^1^ Recent studies reported that children with multiple exposures to general anesthesia are at an increased risk of developing learning disabilities.^2^ Preclinical studies also indicated that neonatal rat exposure to sevoflurane exerted irreversible effects on the development of the central nervous system at both the cellular and systemic levels,^3^, ^4^, ^5^ resulting in long‐term alterations in cognition behavior.^6^ However, the underlying mechanisms as such remain elusive.

The hippocampal CA3 region serves as a nexus of information processing of spatiotemporal memory, which is essential for the storage and retrieval of information.^7^, ^8^ The processing of information in the hippocampus depends on the balance of excitatory and inhibitory transmission.^9^ In the hippocampal CA3, the inhibitory interneurons participate in the performance of cognitive functions by regulating the balance of synaptic excitation/inhibition and neuronal synchronization.^10^, ^11^ More importantly, PV interneurons are one of the most important GABAergic inhibitory interneurons in the hippocampus^12^ and play an important role in the production of gamma oscillations.^13^ Accumulating evidence shows that the dysfunction of PV interneurons resulting in disturbed LFP‐gamma oscillations is associated with the pathogenesis of cognitive disorders.^14^, ^15^, ^16^ However, the role of PV interneurons associated with LFP‐gamma oscillations in the hippocampus in sevoflurane‐induced cognitive impairment in neonates is still unknown.

Synaptic cell adhesion molecule 1 (SynCAM1) is a member of the immunoglobulin superfamily, which mainly expresses on pre‐ and postsynaptic plasma membranes and mediates cell–cell adhesion by trans‐homophilic binding^17^, ^18^ in the developing and mature brains.^19^ Previous studies reported that synCAM1 regulates multiple aspects of neurodevelopment, including synapse formation^20^ and axon‐dendritic contacts.^21^ Interestingly, synCAM1 is specifically expressed in the PV interneurons in the hippocampus.^22^ SynCAM1 is required for the formation and maintenance of excitatory mossy fiber inputs onto pyramidal neurons and PV interneurons in the hippocampal CA3 and their impairments lead to a reduction in excitatory mossy fiber inputs.^23^ SynCAM1 can interact with 4.1B to recruit AMPARs and NMDARs to the postsynaptic membrane and is critical for excitatory transmission in the hippocampus.^24^ SynCAM1 is a key molecule in regulating neuroplasticity and is involved in the pathogenesis of neuropsychiatric disorders.^25^ However, whether the synCAM1‐dependent molecular mechanisms underlying excitatory synapses in PV interneurons contribute to sevoflurane‐induced cognitive impairments remains unknown. In this study, we aimed to investigate whether the synCAM1 in PV interneurons is involved in the pathophysiology of sevoflurane‐induced cognitive impairment and further elucidate whether environmental enrichment^26^ has any potential therapeutic effects on the changed induced by sevoflurane and underlying mechanisms in neonatal rats.

## 2 MATERIALS AND METHODS

### 2.1 Animals

All animal procedures were approved by the Animal Care and Use Committee of the First Affiliated Hospital of Zhengzhou University, Zhengzhou, China. Sprague‐Dawley pregnant rats were purchased from Charles River (Beijing, China). They were housed in the standard laboratory conditions (12‐h light/dark cycle, lights on from 8:00 a.m. to 8:00 p.m.) and temperature (23–24°C) with free access to food and water. Within 24 h of delivery, extra litters were culled, and 12 pups/dam were allowed to grow for the various experiments described below. To control experimental variability, different pups from different dams were used for each set of experiments.

### 2.2 Sevoflurane exposure

The P6 male pups received with (the sevoflurane group) or without (the control groups) 3% sevoflurane and 30% oxygen balanced with air for 2 h a day for 3 consecutive days in the anesthetizing chamber with a temperature maintained at 37 ± 0.5°C. The oxygen concentration in the chamber was measured continuously with a gas analyzer (GE Datex‐Ohmeda, Tewksbury, MA). They were returned to their mothers after their righting reflex recovered upon the termination of sevoflurane exposure at each time.

### 2.3 Enriched environment

After the above treatments, some cohorts were randomly assigned to enriched environment (EE) cages or standard environment (SE) cages. The EE cage (60 × 40 × 20 cm) was equipped with different playing toys, including the running wheel, platforms, tube mazes, and the different combinations of these toys were changed every 3 days. The rats (P10 to P21) with their mother or without mother (P22 to P35) stayed in the EE cages for 2–4 hours per day, while rats in the SE groups were kept in identical cages without toys as the EE group.

### 2.4 Behavioral testing

#### 2.4.1 Y maze test (YMT)

The rats with or without treatments described above at age P35 were subjected to Y maze test.^27^ Briefly, they were placed in the central area and allowed to explore the arms freely under a video‐tracking recording system for 8 min. The sequence and number of arm entries were scored by a researcher who was blinded to the experimental protocol. A spontaneous alternation was defined as a mouse entering all three arms on consecutive choices (ABC, BAC, or CBA but not BAB, CCA, or ABB). The percentage of spontaneous alternation was calculated as: % spontaneous alternation = number of spontaneous alternations/(total number of arm entries‐2) × 100. A high percentage indicates as a good working memory.

#### 2.4.2 Morris water maze (MWM) test

The rats with or without the treatments described above at age P36 were subjected to Morris water maze (MWM) test. The apparatus consisted of a circular tank (150 cm diameter, 55 cm height) filled with opaque water (temperature 23 ± 1°C). The wall of the tank had four visual cues, and the tank was divided into four equal quadrants. A 10 ‐cm diameter platform (as the escape platform) was submerged 1.5 cm below the surface of the water at a constant location in the targeted quadrant. Each rat was tested four times per day from P36 to P40; the rats were placed into the tank from four random points of the tank and were allowed to search for the escape platform for the 90s. Each rat found the platform and was allowed to stay on it for 15 s. If the rat failed to find for escape platform for the 90s, the rat was then guided to the platform and allowed to stay on the platform for 15 s. On P41, the platform was removed, and the rat was allowed to swim freely for 90s. The time spent in the targeted quadrant and the number of platform crossings were recorded by a video camera and analyzed by SMART 3.0 software (Panlab S.L.U.).

### 2.5 Western blot

Hippocampus tissues were harvested at ages P14, 21, 28, 35, and 42 and then homogenized using RIPA lysis buffer (CW2333s, CW, biotech), supplemented with 0.1 mM phenylmethylsulphonyl fluoride (PMSF)‐protease inhibitors (CW2200S, CW, biotech). Protein concentration was determined by a BCA protein assay kit (GK10009, GLPBIO). Equal amounts of protein samples (20 μg) were loaded and separated by 10% or 12% sodium dodecyl sulfate‐polyacrylamide gel electrophoresis (G2037‐50T, Servicebio) transferred onto PVDF membranes (Millipore, Bedford, MA, USA). After blocking with 3% non‐fat milk for 1 h at room temperature, membranes were incubated at 4°C overnight with primary antibodies of chicken anti‐SynCAM1(1:1000, CM004‐3, MBL), rabbit anti‐PV (1:500, A13538, ABclonal), or rat anti‐β‐actin (1:10000, 660009‐1, Proteintech). After washing with Tris Buffer Saline with Tween‐20 (TBST), the membranes were incubated for 2 h at room temperature with secondary antibodies of goat anti‐rabbit IgG (1:10,000, SA00001‐2, Proteintech), goat anti‐mouse IgG (1:10,000, SA00001‐2, Proteintech), and goat anti‐chicken IgG (1:5000, L35001, Signalway) and visualized with an enhanced chemiluminescent detection (ECL) kit (WBKLS0100, Millipore) and analyzed with Image J software.

### 2.6 Immunofluorescence

The rats at ages P35 and 42 were anesthetized with sevoflurane and transcardially perfused with phosphate‐buffered saline (PBS), followed by 4% paraformaldehyde (PFA) in PBS, and their brains were harvested and post‐fixed in 4% PFA in PBS at 4°C for 4 h, then dehydrated in 20%, 30%, and 40% sucrose in PBS at 4°C, and then cut to be coronal sections (30 μm) on a cryostat (CM3050S, Leica). After washing with PBS, the sections were blocked with PBS containing 5% normal goat serum and 0.3% Triton X‐100 for 1 h at room temperature and then incubated overnight at 4°C with primary antibodies: chicken anti‐synCAM1 (1:1000, CM004‐3, MBL), rabbit anti‐PV (1:400, ab181086, Abcam), mouse anti‐PV (1:400, MAB1572, Sigma), mouse anti‐GAD67 (1:400, ab26116, Abcam), guinea pig anti‐Bassoon (1:500, 141004, Synaptic Systems), rabbit anti‐Homer1 (1:500, 160003, Synaptic Systems) or mouse anti‐vGat (1:400, 131011, Synaptic Systems), rabbit anti‐SST (1:500, GTX133119, GeneTex), and rabbit anti‐VIP (1:500, 63269, Cell signaling). The sections were washed with PBS and incubated at room temperature for 2 h with goat anti‐chicken FITC (1:500, L35065, Signalway), goat anti‐rabbit Cy3 (1:200, 111‐165‐003, Jackson ImmunoResearch), goat anti‐mouse TRITC (1:200, 115‐025‐003, Jackson ImmunoResearch), goat anti‐rabbit FITC (1:100, 111‐095‐003, Jackson ImmunoResearch), goat anti‐mouse FITC (1:100, 115‐095‐003, Jackson ImmunoResearch), goat anti‐guinea pig FITC (1:100, 106‐095‐003, Jackson ImmunoResearch), or goat anti‐mouse AMCA (1:50, SA00010‐1, Proteintech). Images were captured by a confocal microscope (A1 MP+, Nikon).

### 2.7 Quantitative RT‐PCR

Hippocampus tissues were harvested at ages P14, 21, 28, 35, and 42. Total RNA of the hippocampus was isolated using the RNA simple Total RNA Kit (DP419, TIANGEN Biochemical Technology), reverse transcription using the Primescript RT reagent kit with gDNA Eraser (RR420A, TaKaRa), and quantitative real‐time PCR using the TB Premix Ex Taq (RR420A, TaKaRa) were done (Biosystems StepOnePlus Real‐Time PCR System). Analysis was performed using the relative standard curve method with GAPDH as the calibration gene for normalization. Primer sequences (forward, reverse) were as follows: SynCAM1 (5′‐AGTGGGGAAAGCTCATTCGG‐3′, 5′ ‐ACTGTCCAGTTCTTCTGCGG‐3′), PV (5′‐CGCCCTGATATTTCCTGCCT‐3′, 5′ ‐CCCCTTCCGTTCTGCTCTTT‐3′), GAPDH (5′‐GCATCTTCTTGTGCAGTGCC‐3′, 5′‐GATGGTGATGGGTTTCCCGT‐3′).

### 2.8 Golgi staining

The rats at age P42 were deeply anesthetized by sevoflurane and rapidly sacrificed. The brains were removed from the skull as quickly as possible for Golgi staining (Golgi Stain Kit (#PK401, FD NeuroTechnologies, INC) accordingly.^28^ Pyramidal cells in CA3 were captured with a confocal microscope at 20 × and 100 × objectives (Olympus FluoView FV1000, Tokyo, Japan). Their morphology and dendritic spines were analyzed with Neuron J plugin, Sholl analyses, and Cell Counter plugin with Image J software.

### 2.9 Stereotaxic microinjection

After being exposed to sevoflurane treatments, P9 pups were anesthetized and fixed on the stereotaxic instrument (RWD, Shenzhen, China). Four hundred nL of AAV‐PV‐Cre and AAV‐ef1a‐DIO‐synCAM1‐2A‐mcherry‐WPRE‐PA or AAV‐ef1a‐DIO‐mcherry‐WPRE‐PA (Brain VTA Technology Corporation, Wuhan) were injected bilaterally into the dorsal hippocampal CA3 region (anteroposterior(AP), −2.05 mm; mediolateral(ML), ±3.05 mm; dorsoventral(DV), −2.56 mm) at a rate of 40 nL/min using a 5 μL microsyringe (RWD Life Science Co, Shenzhen, China), After injection, the needle was maintained at the injection site for 10 min and they then returned to their home cage with dams after gaining full recovery.

### 2.10 Chemogenetic manipulations

After exposed to sevoflurane treatments, the P9 pups received stereotaxic hippocampal CA3 injections of AAV‐PV‐Cre and AAV‐DIO‐hM3Dq‐mCherry or AAV‐DIO‐mCherry (Brain VTA Technology Corporation, Wuhan). Twenty‐one days after viral injection, clozapine‐N‐oxide (CNO, 3 mg/kg in 1% DMSO, MCE, China, HY‐17366) or vehicle was injected intraperitoneally and YMT and MWM test were performed 45 min after injection.

### 2.11 In vivo electrophysiology

The rats at age P30 were anesthetized with 1.5% sevoflurane in O_2_ and craniotomies were performed under stereotaxic guidance. Briefly, after balancing the skull, a rectangular craniotomy (5 mm × 4 mm) was performed to expose skull and the dura was opened. An eight‐channel microelectrode (Kedou Brain‐computer Technology Co., Ltd., Suzhou, China) was implanted into the dorsal hippocampal CA3 (AP—3.34 mm; ML ± 3.48 mm; DV—3.42 mm). Two screws were fixed on the skull and attached to stainless steel wires as ground. After 5‐day recovery period, rats are placed in an open field chamber and move freely and local field potential (LFP) were recorded by the Apollo II 32ch digital signal processor (Bio‐Signal Technologies, Nanjing, China) and amplified, filtered at a 300–5000 Hz bandwidth. In our study, the bands were divided into slow gamma (30–50 Hz) and fast gamma (65–120 Hz) and all data were analyzed with Neuroexplorer 5 (Plexon Inc., Dallas, TX) software.

### 2.12 Statistical analysis

Data are expressed as mean ± SEM and analyzed with GraphPad Prism 7.0. All data were tested by Shapiro‐Wilk or Kolmogorov‐Smirnov normality test. Data followed a normal distribution and were analyzed using two‐tailed Student's *T*‐test, two‐way ANOVA with Tukey's post hoc test. Otherwise, data were analyzed using the Mann‐Whitney test. The MWM test escape latency data were analyzed by two‐way repeated‐measures ANOVA followed by Bonferroni's post hoc test. Difference was considered to be significant at *p* < 0.05.

## 3 RESULTS

### 3.1 Sevoflurane exposure induced cognitive impairments

**FIGURE 1:**
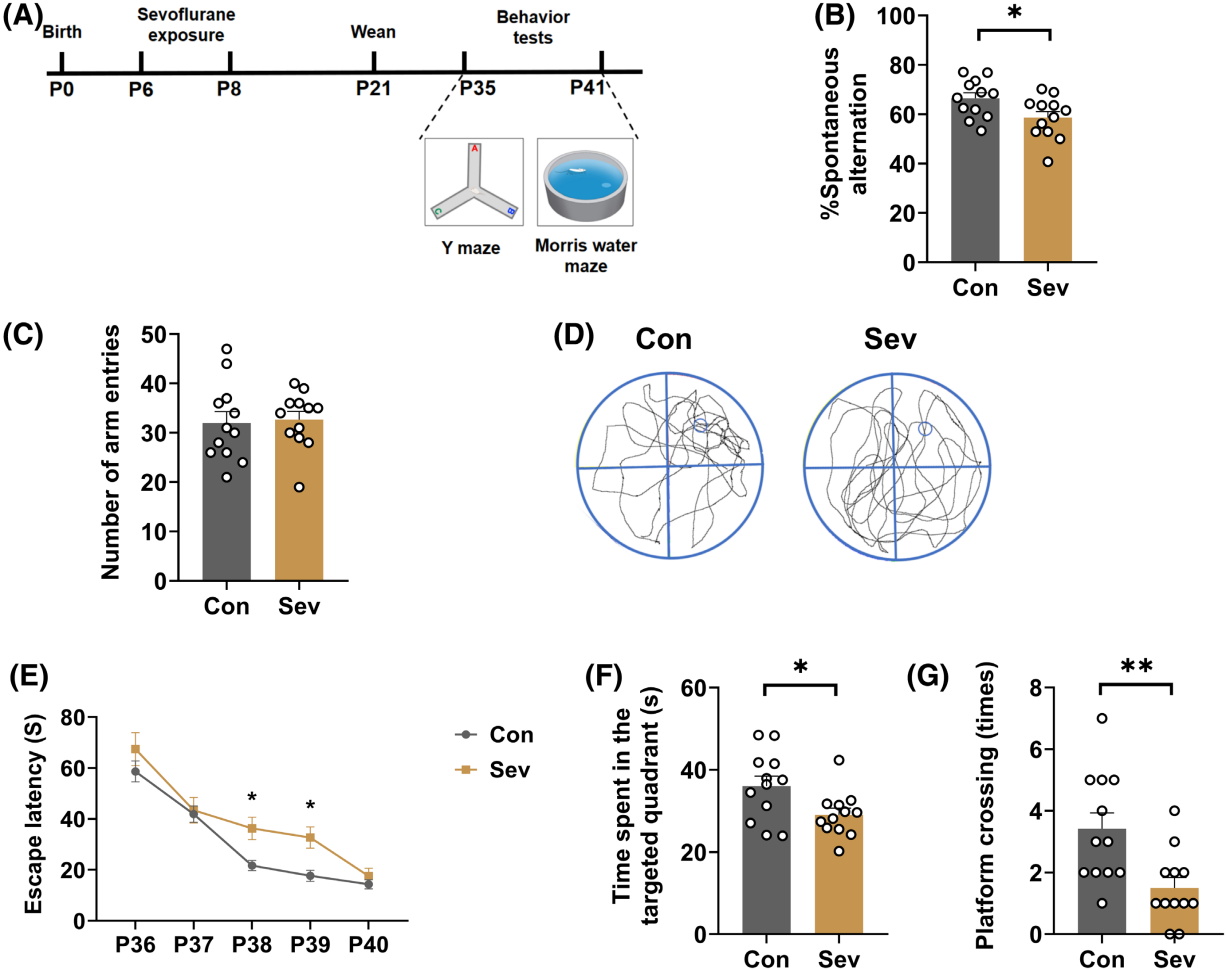
Sevoflurane exposure induced cognitive impairments. (A) Flowchart of behavior tests after control or sevoflurane exposure. (B) Spontaneous alternation (*t*_(22)_ = 2.341, *p* = 0.0287) and (C) number of arm entries of YMT (*t*_(22)_ = 0.2361, *p* = 0.8156) in control and sevoflurane‐exposed rats. (D) Representative swimming tracks of the control and sevoflurane‐exposed rats during the probe trial of the MWM test. (E) Escape latency (Interaction, *F*_(4,88)_ = 1.508, *p* = 0.2067; Time, *F*_(3.265,51.69)_ = 51.69, *p* < 0.0001; Treatment, *F*_(1,22)_ = 7.732, *p* = 0.0109), (F) time spent in the target quadrant (*t*_(22)_ = 2.424, *p* = 0.024), and (G) platform crossing times of the MWM test (*t*_(22)_ = 3.117, *p* = 0.005) in control and sevoflurane‐exposed rats. Data are expressed as mean ± SEM (*n* = 12 rats/group). The MWM test escape latency data were analyzed by two‐way repeated‐measures ANOVA followed by Bonferroni's post hoc test, and other results were analyzed by unpaired student's *t*‐test. *p < 0.05, **p < 0.01 vs. the control group. Con, control; MWM, Morris water maze; Sev, sevoflurane; YMT, Y maze test.

P6 rat pups were exposed to 3% sevoflurane for 2 h daily for 3 days and the working memory and spatial learning test with the YMT and MWM test were performed from P35 to P41 (Figure 1A).

In the YMT, the sevoflurane‐exposed group had less spontaneous alternation compared with the control group (*t*_(22)_ = 2.341, *p* = 0.0287; Figure 1B), and no differences in the total number of arm entries were observed between the two groups (*t*_(22)_ = 0.2361, *p* = 0.8156; Figure 1C). In the MWM test, the escape latency was shorter in the sevoflurane‐exposed group compared with the control group on P38 and P39 (Interaction: *F*_(4,88)_ = 1.508, *p* = 0.2067; Time: *F*_(3.265,51.69)_ = 51.69, *p* < 0.0001; Treatment: *F*_(1,22)_ = 7.732, *p* = 0.0109; Figure 1D,E). In addition, the time in the target quadrant (*t*_(22)_ = 2.424, *p* = 0.024; Figure 1F) and the number of platform crossing (*t*_(22)_ = 3.117, *p* = 0.005; Figure 1G) was lower in the sevoflurane‐exposed group compared with the control group on P41.

### 3.2 Sevoflurane exposure decreased PV phenotype in the hippocampal CA3 region

**FIGURE 2:**
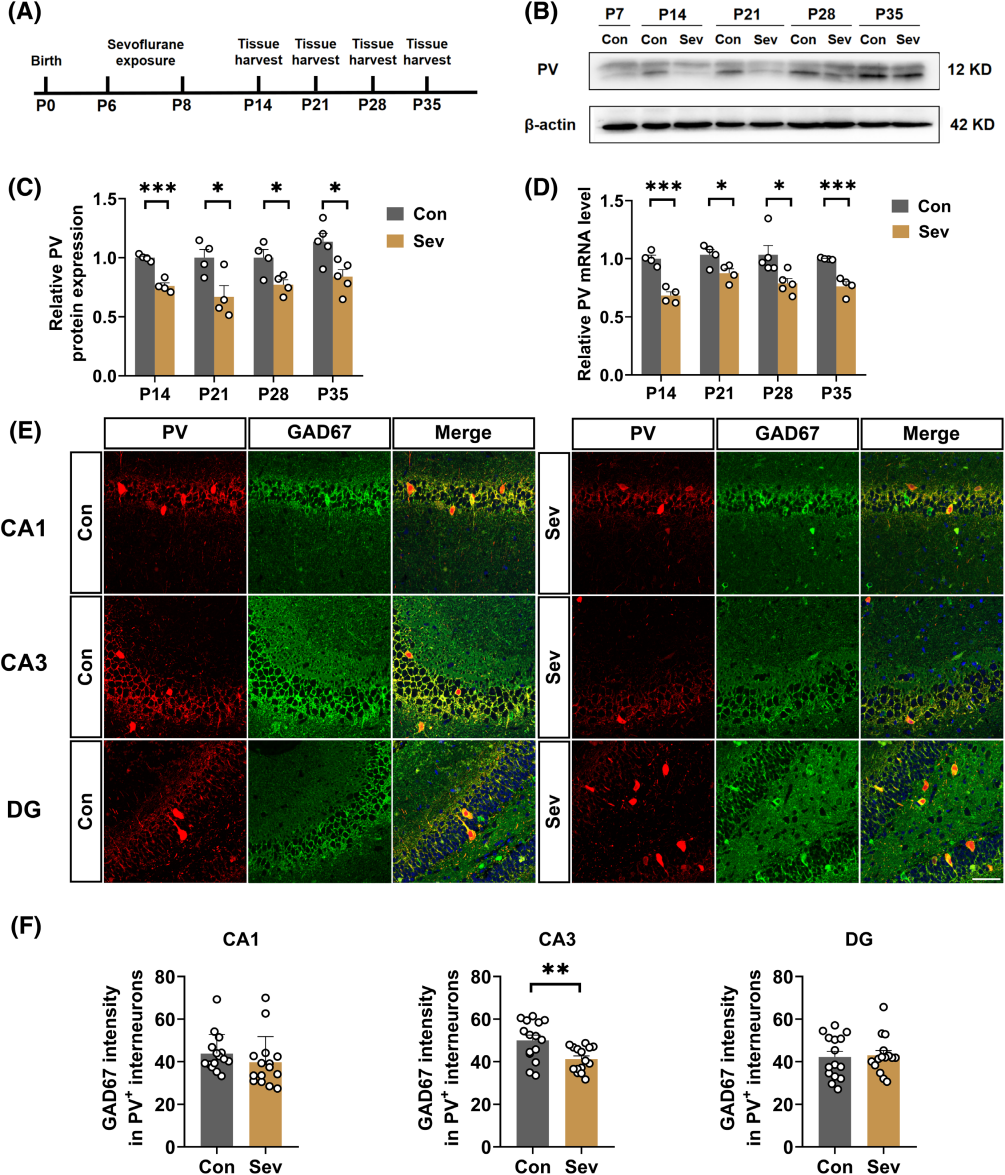
Sevoflurane exposure decreased PV phenotype in the hippocampal CA3 region (A) Flowchart of biochemistry tests after control or sevoflurane exposure. (B) Representative western blots bands of PV in the hippocampus. (C) Quantitative analysis of PV protein level in the hippocampus of control and sevoflurane‐exposed rats on P14 (*t*_(6)_ = 8.054, *p* = 0.0002), P21(*t*_(6)_ = 2.791, *p* = 0.0315), P28 (*t*_(6)_ = 2.822, *p* = 0.0303), and P35 (*t*_(8)_ = 3.193, *p* = 0.0128). (D) Quantitative analysis of PV mRNA level in the hippocampus of control and sevoflurane‐exposed rats on P14 (*t*_(6)_ = 7.109, *p* = 0.0004), P21 (*t*_(6)_ = 2.648, *p* = 0.0381), P28 (*t*_(8)_ = 2.726, *p* = 0.0260), and P35 (*t*_(6)_ = 6.072, *p* = 0.0009). (E) Representative images of double‐labeling immunostaining of PV (red) and GAD67 (green) in the hippocampus. Scale bar: 100 μm. (F) Quantitative GAD67 immunofluorescent intensity in PV interneurons in the hippocampal CA1 (*t*_(28)_ = 1.045, *p* = 0.3050), CA3 (*t*_(28)_ = 3.123, *p* = 0.0041), and DG (*t*_(28)_ = 0.2207, *p* = 0.8269) of control and sevoflurane‐exposed rats on P35. Data are expressed as mean ± SEM (*n* = 3–5 rats/group). Results were analyzed by unpaired student's *t*‐test. *p < 0.05, **p < 0.01, ***p < 0.001 vs. the control group. Con, control; PV, parvalbumin; Sev, sevoflurane.

To explore the potential mechanisms of sevoflurane‐induced cognitive impairment, we evaluated the expression of PV in the developing hippocampus in the control group and the sevoflurane‐exposed group (Figure 2A). The data from western blot and qPCR showed that sevoflurane exposure decreased the protein and mRNA level of PV in the hippocampus compared with the control group on P14, 21, 28, and 35 (PV protein level: P14 (*t*_(6)_ = 8.054, *p* = 0.0002), P21(*t*_(6)_ = 2.791, *p* = 0.0315), P28 (*t*_(6)_ = 2.822, *p* = 0.0303), P35 (*t*_(8)_ = 3.193, *p* = 0.0128); Figure 2B,C. PV mRNA level: P14 (*t*_(6)_ = 7.109, *p* = 0.0004), P21 (*t*_(6)_ = 2.648, *p* = 0.0381), P28 (*t*_(8)_ = 2.726, *p* = 0.0260), P35 (*t*_(6)_ = 6.072, *p* = 0.0009); Figure 2D). To examine the PV phenotype, we assessed the immunofluorescent intensity of GAD67 in the PV interneurons, a biomarker for PV interneuron differentiation. The data revealed that sevoflurane exposure decreased the immunofluorescent intensity of GAD67 in the PV interneurons in the hippocampal CA3 on P35, with no changes observed in the CA1 and DG regions (CA1 (*t*_(28)_ = 1.045, *p* = 0.3050), CA3 (*t*_(28)_ = 3.123, *p* = 0.0041), DG (*t*_(28)_ = 0.2207, *p* = 0.8269); Figure 2E,F).

### 3.3 Chemogenetic activation of PV interneurons in the hippocampal CA3 reversed sevoflurane‐induced cognitive impairments

**FIGURE 3:**
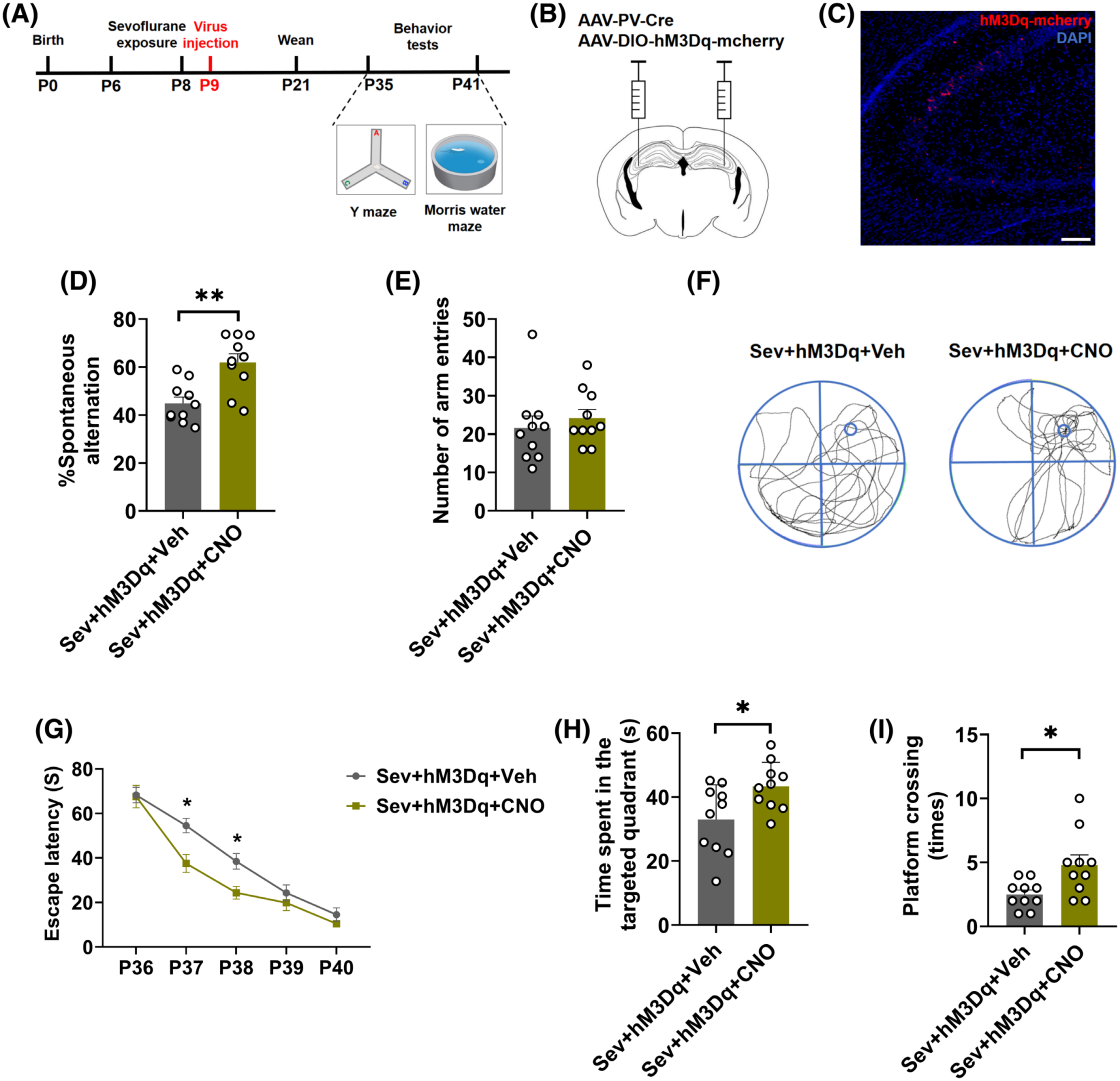
Chemogenetic activation of PV interneurons in the hippocampal CA3 reversed sevoflurane‐induced cognitive impairments. (A) Flowchart of virus injection and behavior tests. (B) Schematic showing virus injection of AAV‐PV‐cre and AAV‐DIO‐hM3Dq‐mCherry or AAV‐DIO‐mCherry into the hippocampal CA3. (C) Representative images showing virus expression within the hippocampal CA3. Scar bar = 200 μm. (D) Spontaneous alternation (*t*_(18)_ = 3.812, *p* = 0.0013) and (E) number of arm entries of YMT (*t*_(18)_ = 0.6774, *p* = 0.5068) in CNO‐treated hM3Dq rats or vehicle‐treated hM3Dq rats. (F) Representative swimming tracks of CNO‐treated hM3Dq rats or vehicle‐treated hM3Dq rats during the probe trial of the MWM test. (G) Escape latency (Interaction, *F*_(4,72)_ = 3.723, *p* = 0.0082; Time, *F*_(3.060,55.09)_ = 140, *p* < 0.0001; Treatment, *F*_(1,18)_ = 4.879, *p* = 0.0404), (H) time spent in the target quadrant (*t*_(18)_ = 2.500, *p* = 0.0223) and (I) platform crossing times of the MWM test (*t*_(18)_ = 2.644, *p* = 0.0165) in CNO‐treated hM3Dq rats or vehicle‐treated hM3Dq rats. Data are expressed as mean ± SEM (*n* = 10 rats/group). The MWM test escape latency data were analyzed by a two‐way repeated‐measures ANOVA followed by Bonferroni's post hoc test; other results were analyzed by an unpaired student's *t*‐test. *p < 0.05, **p < 0.01 vs. the control group. CNO, clozapine N‐oxide; MWM, Morris water maze; Sev, sevoflurane; Veh, vehicle; YMT, Y maze test.

To determine the role of PV interneurons in the hippocampal CA3 on sevoflurane‐induced cognitive impairments, we used a chemogenetic manipulation to specifically regulate the activity of PV interneurons and conducted the YMT and MWM tests (Figure 3A,B). Immunofluorescence confirmed that the virus was successfully injected into the hippocampal CA3 and stably transfected (Figure 3C). In the YMT, chemogenetic activation of PV interneurons in the hippocampal CA3 increased spontaneous alternation (*t*_(18)_ = 3.812, *p* = 0.0013; Figure 3D), while there were no differences in the total number of arm entries between two groups (*t*_(18)_ = 0.6774, *p* = 0.5068; Figure 3E). In the MWM test, chemogenetic activation of PV interneurons in the hippocampal CA3 induced a drop in the escape latency on P37 and P38 (Interaction: *F*_(4,72)_ = 3.723, *p* = 0.0082; Time: *F*_(3.060,55.09)_ = 140, *p* < 0.0001; Treatment: *F*_(1,18)_ = 4.879, *p* = 0.0404; Figure 3F,G), increased the time spent in the target quadrant (*t*_(18)_ = 2.500, *p* = 0.0223; Figure 3H) and platform crossing times compared with the sevoflurane‐exposed group (*t*_(18)_ = 2.644, *p* = 0.0165; Figure 3I). These observations suggested that PV interneurons are involved in sevoflurane‐induced cognitive impairments.

### 3.4 Sevoflurane decreased synCAM1 expression in PV interneurons in the hippocampal CA3 region

**FIGURE 4:**
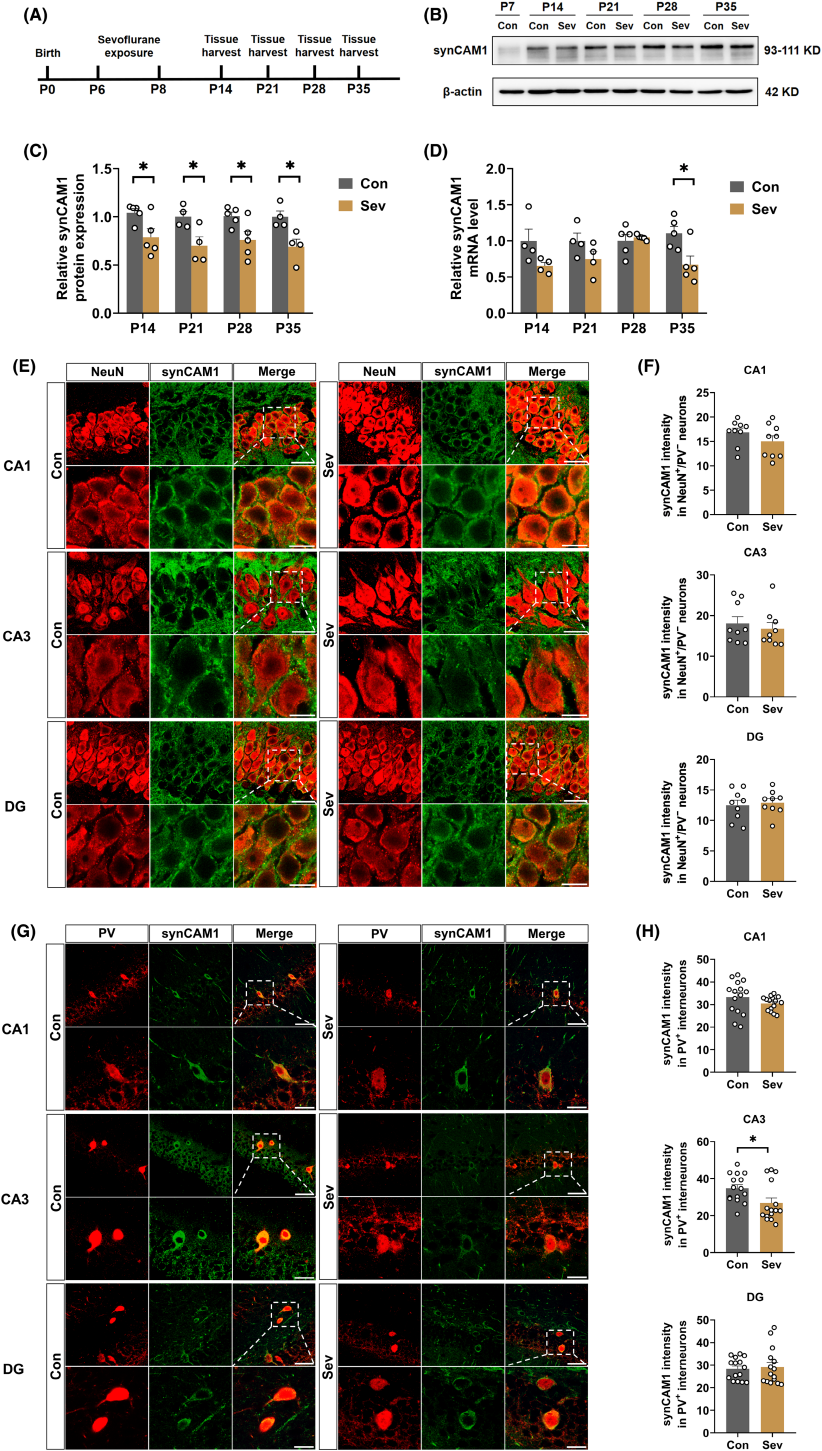
Sevoflurane decreased synCAM1 expression in PV interneurons in the hippocampal CA3 region (A) Flowchart of biochemistry tests after control or sevoflurane exposure. (B) Representative western blots bands of synCAM1 in the hippocampus. (C) Quantitative analysis of synCAM1 protein level in the hippocampus of control and sevoflurane‐exposed rats on P14 (*t*_(8)_ = 2.584, *p* = 0.0324), P21 (*t*_(6)_ = 2.797, *p* = 0.0313), P28 (*t*_(8)_ = 2.555, *p* = 0.0339), and P35 (*t*_(6)_ = 3.157, *p* = 0.0196). (D) Quantitative analysis of synCAM1 mRNA level in the hippocampus of control and sevoflurane‐exposed rats on P14 (*t*_(6)_ = 2.027, *p* = 0.0890), P21 (*t*_(6)_ = 1.636, *p* = 0.153), P28 (*t*_(8)_ = 0.5006, *p* = 0.6301), and P35 (*t*_(8)_ = 2.840, *p* = 0.0218). (E) Representative images of double‐labeling immunostaining of NeuN^+^/PV^−^ (red) and synCAM1 (green) in the hippocampus. Scale bar: 20 μm. (F) Quantitative synCAM1 immunofluorescent intensity in NeuN^+^/PV^−^ neurons in the hippocampal CA1 (*t*_(16)_ = 1.279, *p* = 0.2193), CA3 (*t*_(16)_ = 0.5856, *p* = 0.5663), and DG (*t*_(16)_ = 0.4209, *p* = 0.6794) of control and sevoflurane‐exposed rats on P35. (G) Representative images of double‐labeling immunostaining of PV (red) and synCAM1 (green) in the hippocampus. Scale bar: 50 μm (overview), 20 μm (zoom). (H) Quantitative synCAM1 immunofluorescent intensity in PV interneurons in the hippocampal CA1 (*t*_(28)_ = 1.376, *p* = 0.1798), CA3 (U = 59, *p* = 0.026), and DG (*t*_(28)_ = 0.3225, *p* = 0.7495) of control and sevoflurane‐exposed rats on P35. Data are expressed as mean ± SEM (*n* = 3–5 rats/group). Results were analyzed by unpaired student's *t*‐test or Mann–Whitney test. *p < 0.05 vs. the control group. Con, control; Sev, sevoflurane.

Moreover, neonatal rats exposed to sevoflurane exhibited decreased expression of synCAM1 in the PV interneurons in the hippocampal CA3. Given the importance of synCAM1 in the PV interneurons, we examined its expression in the developing hippocampus to determine the mechanisms underlying the loss of PV phenotype in the sevoflurane‐exposed group (Figure 4A). Sevoflurane exposure decreased the protein level of synCAM1 compared with that in the control group on P14 (*t*_(8)_ = 2.584, *p* = 0.0324), 21 (*t*_(6)_ = 2.797, *p* = 0.0313), 28 (*t*_(8)_ = 2.555, *p* = 0.0339), and 35 (*t*_(6)_ = 3.157, *p* = 0.0196) (Figure 4B,C), while a decrease in mRNA level was observed only on P35 (*t*_(8)_ = 2.840, *p* = 0.0218), with no changes on P14 (*t*_(6)_ = 2.027, *p* = 0.0890), 21 (*t*_(6)_ = 1.636, *p* = 0.153), and 28 (*t*_(8)_ = 0.5006, *p* = 0.6301) (Figure 4D). Double‐labeling immunostaining results revealed that there were no significant differences in the immunofluorescent intensity of synCAM1 in the NeuN+/PV‐ neurons in the hippocampal CA1, CA3, and DG between the two groups (CA1 (*t*_(16)_ = 1.279, *p* = 0.2193), CA3 (*t*_(16)_ = 0.5856, *p* = 0.5663), DG (*t*_(16)_ = 0.4209, *p* = 0.6794); Figure 4E,F). However, the immunofluorescent intensity of synCAM1 in the PV+ interneurons was decreased in the hippocampal CA3 on P35, with no changes in the CA1 and DG regions (CA1 (*t*_(28)_ = 1.376, *p* = 0.1798), CA3 (U = 59, *p* = 0.026), and DG (*t*_(28)_ = 0.3225, *p* = 0.7495; Figure 4G,). In addition, we found there were no significant differences in the immunofluorescent intensity of synCAM1 in the SST and VIP interneurons in the hippocampal CA1, CA3, and DG between the two groups (SST: CA1 (*t*_(28)_ = 1.588, *p* = 0.8750), CA3 (*t*_(28)_ = 0.7757, *p* = 0.4444), DG (*t*_(28)_ = 0.1.225, *p* = 0.2308); VIP: CA1 (*t*_(28)_ = 0.1121, *p* = 0.9115), CA3 (*t*_(28)_ = 0.6293, *p* = 0.5343), and DG (*t*_(28)_ = 1.217, *p* = 0.2337); Figure S1A–D). These data suggested that decreased synCAM1 expression induced by sevoflurane exposure was most prominent in the hippocampal CA3 PV interneurons.

### 3.5 SynCAM1 in PV interneurons was involved in the alleviation of sevoflurane‐induced cognitive impairment by EE

**FIGURE 5:**
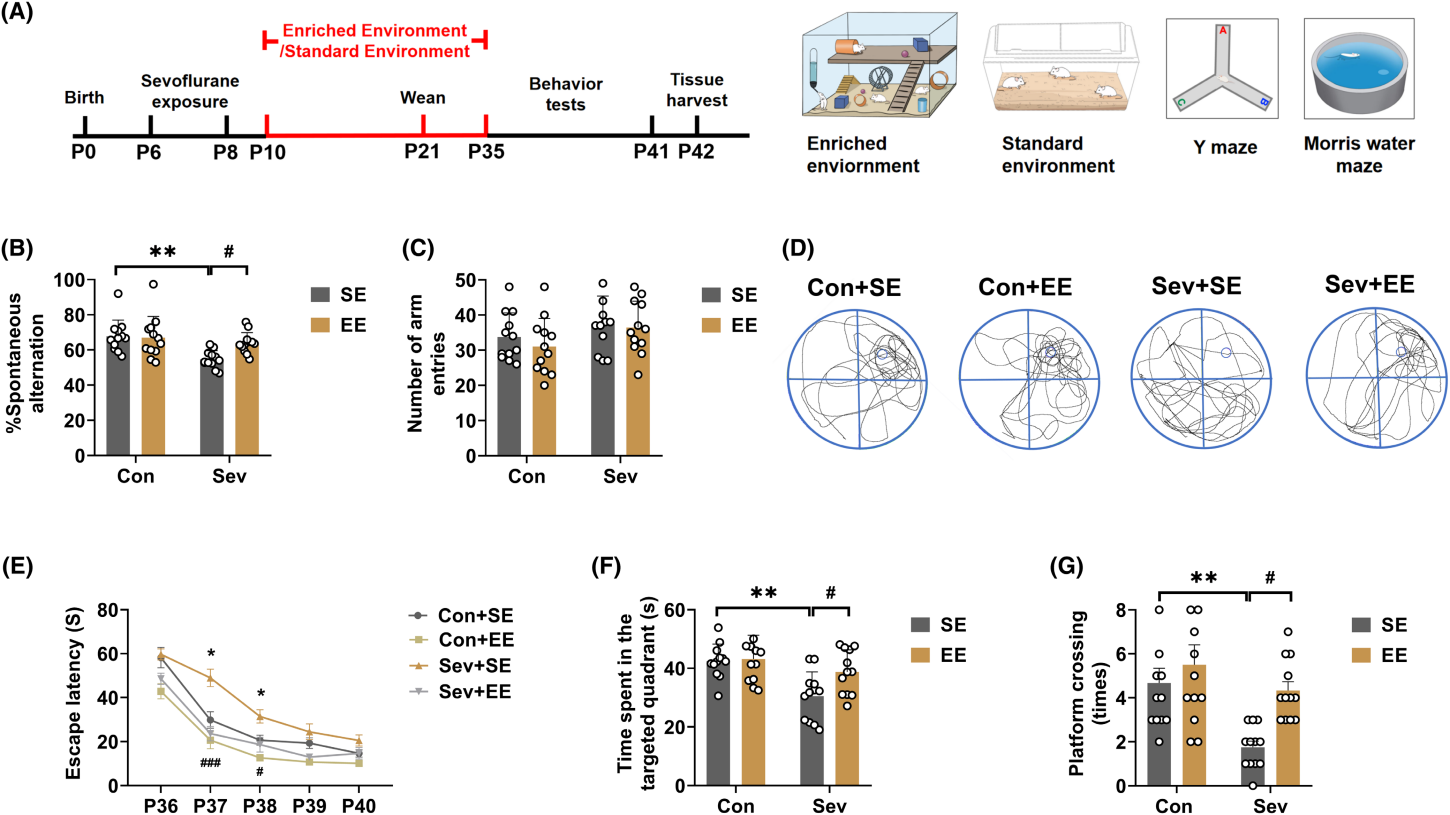
Enriched environment reversed sevoflurane‐induced cognitive impairments. (A) Flowchart of the experimental SE and EE procedure and behavior tests. (B) Spontaneous alternation (Interaction: *F*_(1,44)_ = 4.189, *p* = 0.0467; Sev: *F*_(1,44)_ = 11.08, *p* = 0.0018; EE: *F*_(1,44)_ = 3.044, *p* = 0.0880), and (C) number of arm entries of YMT (Interaction: *F*_(1,44)_ = 0.1443, *p* = 0.7058; Sev: *F*_(1,44)_ = 4.367, *p* = 0.0425; EE: *F*_(1,44)_ = 0.7636, *p* = 0.3869) in control and sevoflurane‐exposed rats treated with SE or EE. (D) Representative swimming tracks of the control and sevoflurane‐exposed rats treated with SE or EE during the probe trial of the MWM test. (E) Escape latency (Interaction, *F*_(12,176)_ = 1.808, *p* = 0.0499; Time, *F*_(3.517,154.8)_ = 115.2, *p* < 0.0001; Treatment, *F*_(3,44)_ = 30.79, *p* < 0.0001), (F) spent time in the target quadrant (Interaction: *F*_(1,44)_ = 3.052, *p* = 0.0876; Sev: *F*_(1,44)_ = 14.22, *p* = 0.0005; EE: *F*_(1,44)_ = 4.296, *p* = 0.0441), and (G) platform crossing times of the MWM test (Interaction: *F*_(1,44)_ = 2.015, *p* = 0.1628; Sev: *F*_(1,44)_ = 10.97, *p* = 0.0019; EE: *F*_(1,44)_ = 7.682, *p* = 0.0081) in control and sevoflurane‐exposed rats treated with SE or EE. Data are expressed as mean ± SEM (*n* = 12 rats/group). The MWM test escape latency data were analyzed by two‐way repeated‐measures ANOVA followed by Tukey post hoc test, other results were analyzed by two‐way ANOVA followed by Tukey post hoc test. *p < 0.05, **p < 0.01, ***p < 0.001 vs. the Con+SE group. #*p* < 0.05, ##*p* < 0.01, ###*p* < 0.001 vs. the Sev + SE group. Con, control; EE, enriched environment; MWM, Morris water maze; SE, standard environment; Sev, sevoflurane; YMT, Y maze test.

**FIGURE 6:**
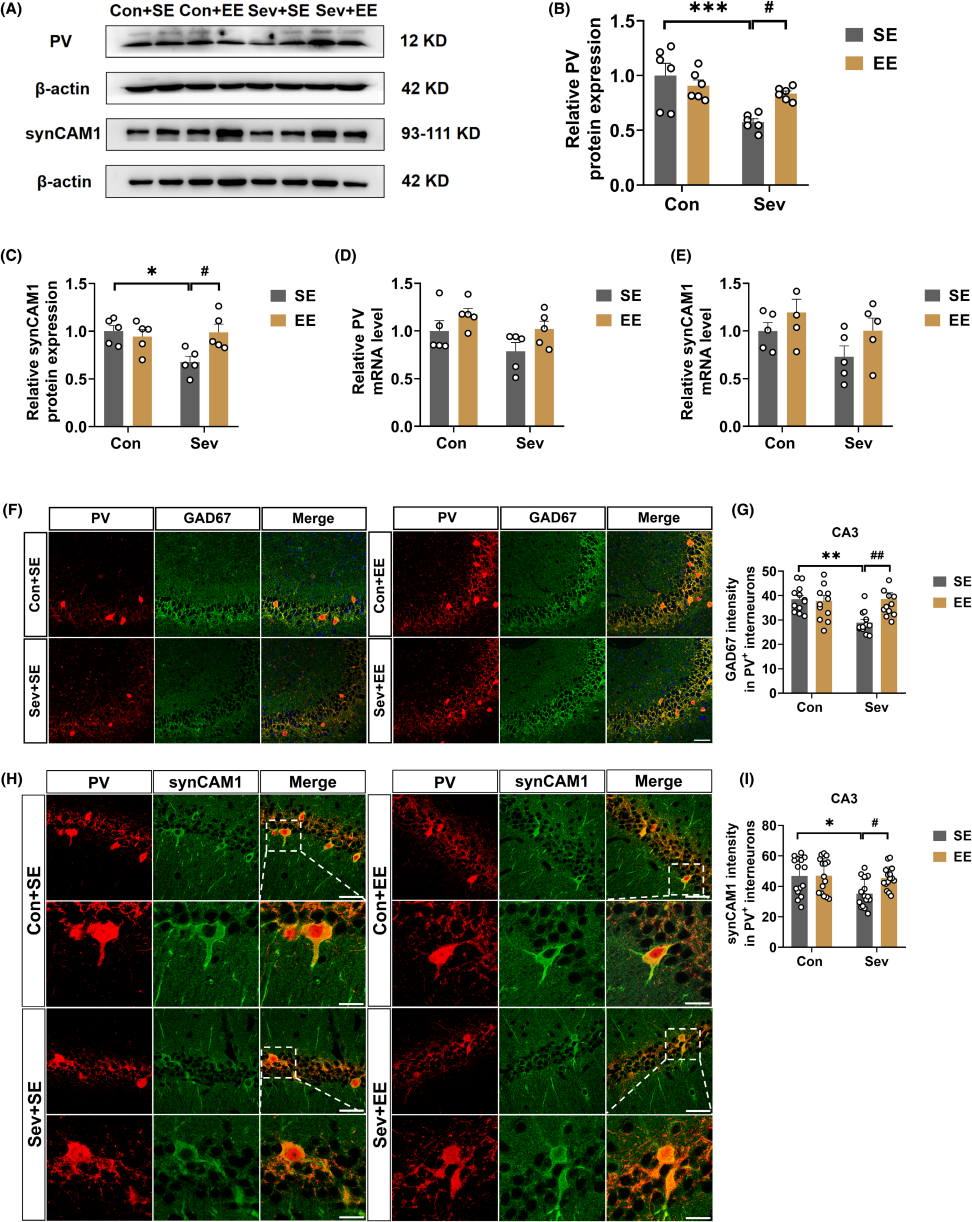
Enriched environment reversed sevoflurane‐induced decreased PV and synCAM1 expression in hippocampal CA3. (A) Representative western blot bands of PV and synCAM1 in the hippocampus. (B, C) Quantitative analysis of PV protein level (Interaction: *F*_(1,20)_ = 7.368, *p* = 0.0133; Sev: *F*_(1,20)_ = 14.49, *p* = 0.0011; EE: *F*_(1,20)_ = 1.642, *p* = 0.2148) and synCAM1 protein level (Interaction: *F*_(1,16)_ = 6.925, *p* = 0.0181; Sev: *F*_(1,16)_ = 3.951, *p* = 0.0642; EE: *F*_(1,16)_ = 3.379, *p* = 0.0847) in the hippocampus of control and sevoflurane‐exposed rats treated with SE or EE. (D, E) Quantitative analysis of PV mRNA level (Interaction: *F*_(1,16)_ = 0.1183, *p* = 0.7354; Sev: *F*_(1,16)_ = 4.310, *p* = 0.0544; EE: *F*_(1,16)_ = 5.314, *p* = 0.0349) and synCAM1 mRNA level (Interaction: *F*_(1,16)_ = 0.1074, *p* = 0.7474; Sev: *F*_(1,16)_ = 3.710, *p* = 0.0720; EE: *F*_(1,16)_ = 3.793, *p* = 0.0692) in the hippocampus of control and sevoflurane‐exposed rats treated with SE or EE. (F) Representative images of double‐labeling immunostaining of PV (red) and GAD67 (green) in the hippocampus. Scale bar: 100 μm. (G) Quantitative GAD67 immunofluorescent intensity in PV interneurons in the hippocampal CA3 of control and sevoflurane‐exposed rats treated with SE or EE (Interaction: *F*_(1,44)_ = 6.936, *p* = 0.0116; Sev: *F*_(1,44)_ = 5.036, *p* = 0.0299; EE: *F*_(1,44)_ = 5.121, *p* = 0.0286). (H) Representative images of double‐labeling immunostaining of PV (red) and synCAM1 (green) in the hippocampus. Scale bar: 50 μm (overview), 20 μm (zoom). (I) Quantitative synCAM1 immunofluorescent intensity in PV interneurons in the hippocampal CA3 of control and sevoflurane‐exposed rats treated with SE or EE. (Interaction: *F*_(1,56)_ = 3.673, *p* = 0.0604; Sev: *F*_(1,56)_ = 6.209, *p* = 0.0157; EE: *F*_(1,56)_ = 3.878, *p* = 0.0539). Data are expressed as mean ± SEM (*n* = 3–6 rats/group). Results were analyzed by two‐way ANOVA followed by the Tukey post hoc test. *p < 0.05, **p < 0.01, ***p < 0.001 vs. the Con+SE group. #*p* < 0.05, ##*p* < 0.01, ###*p* < 0.001 vs. the Sev + SE group. Con, control; EE, enriched environment; PV, parvalbumin; SE, standard environment; Sev, sevoflurane.

Furthermore, synCAM1 in the PV interneurons and PV interneurons themselves were found to be involved in the alleviation of sevoflurane‐induced cognitive impairment by an enriched environment (EE). Therefore, we asked whether EE alleviates the sevoflurane‐induced cognitive impairments exposure evidenced by the YMT and MWM test (Spontaneous alternation: Interaction: *F*_(1,44)_ = 4.189, *p* = 0.0467; Sev: *F*_(1,44)_ = 11.08, *p* = 0.0018; EE: *F*_(1,44)_ = 3.044, *p* = 0.0880; number of arm entries of YMT: Interaction: *F*_(1,44)_ = 0.1443, *p* = 0.7058; Sev: *F*_(1,44)_ = 4.367, *p* = 0.0425; EE: *F*_(1,44)_ = 0.7636, *p* = 0.3869, Escape latency: Interaction: *F*_(12,176)_ = 1.808, *p* = 0.0499; Time: *F*_(3.517,154.8)_ = 115.2, *p* < 0.0001; Treatment: *F*_(3,44)_ = 30.79, *p* < 0.0001; spent time in the target quadrant: Interaction: *F*_(1,44)_ = 3.052, *p* = 0.0876; Sev: *F*_(1,44)_ = 14.22, *p* = 0.0005; EE: *F*_(1,44)_ = 4.296, *p* = 0.0441; platform crossing times: Interaction: *F*_(1,44)_ = 2.015, *p* = 0.1628; Sev: *F*_(1,44)_ = 10.97, *p* = 0.0019; EE: *F*_(1,44)_ = 7.682, *p* = 0.0081; Figure 5A–G). To investigate the potential mechanisms of the alleviation, we evaluated the expression levels of PV and synCAM1 in the control and sevoflurane‐exposed group treated with SE or EE and found that EE reversed the sevoflurane‐induced decreased PV and synCAM1 protein level (PV protein level: Interaction: *F*_(1,20)_ = 7.368, *p* = 0.0133; Sev: *F*_(1,20)_ = 14.49, *p* = 0.0011; EE: *F*_(1,20)_ = 1.642, *p* = 0.2148; synCAM1 protein level: Interaction: *F*_(1,16)_ = 6.925, *p* = 0.0181; Sev: *F*_(1,16)_ = 3.951, *p* = 0.0642; EE: *F*_(1,16)_ = 3.379, *p* = 0.0847; Figure 6A–C), but there were no differences in the mRNA level of PV and synCAM1 among the four groups (PV mRNA level: Interaction: *F*_(1,16)_ = 0.1183, *p* = 0.7354; Sev: *F*_(1,16)_ = 4.310, *p* = 0.0544; EE: *F*_(1,16)_ = 5.314, *p* = 0.0349; synCAM1 mRNA level: Interaction: *F*_(1,16)_ = 0.1074, *p* = 0.7474; Sev: *F*_(1,16)_ = 3.710, *p* = 0.0720; EE: *F*_(1,16)_ = 3.793, *p* = 0.0692; Figure 6D,E). Exposure to the EE reversed the sevoflurane‐induced decreased GAD67 and synCAM1 immunoreactivity in PV interneurons in the hippocampal CA3 (GAD67 immunoreactivity in PV interneurons: Interaction: *F*_(1,44)_ = 6.936, *p* = 0.0116; Sev: *F*_(1,44)_ = 5.036, *p* = 0.0299; EE: *F*_(1,44)_ = 5.121, *p* = 0.0286; synCAM1 immunoreactivity in PV interneurons: Interaction: *F*_(1,56)_ = 3.673, *p* = 0.0604; Sev: *F*_(1,56)_ = 6.209, *p* = 0.0157; EE: *F*_(1,56)_ = 3.878, *p* = 0.0539; Figure 6F–I).

### 3.6 Enriched environments reversed sevoflurane‐induced decreased excitatory input onto PV interneurons

**FIGURE 7:**
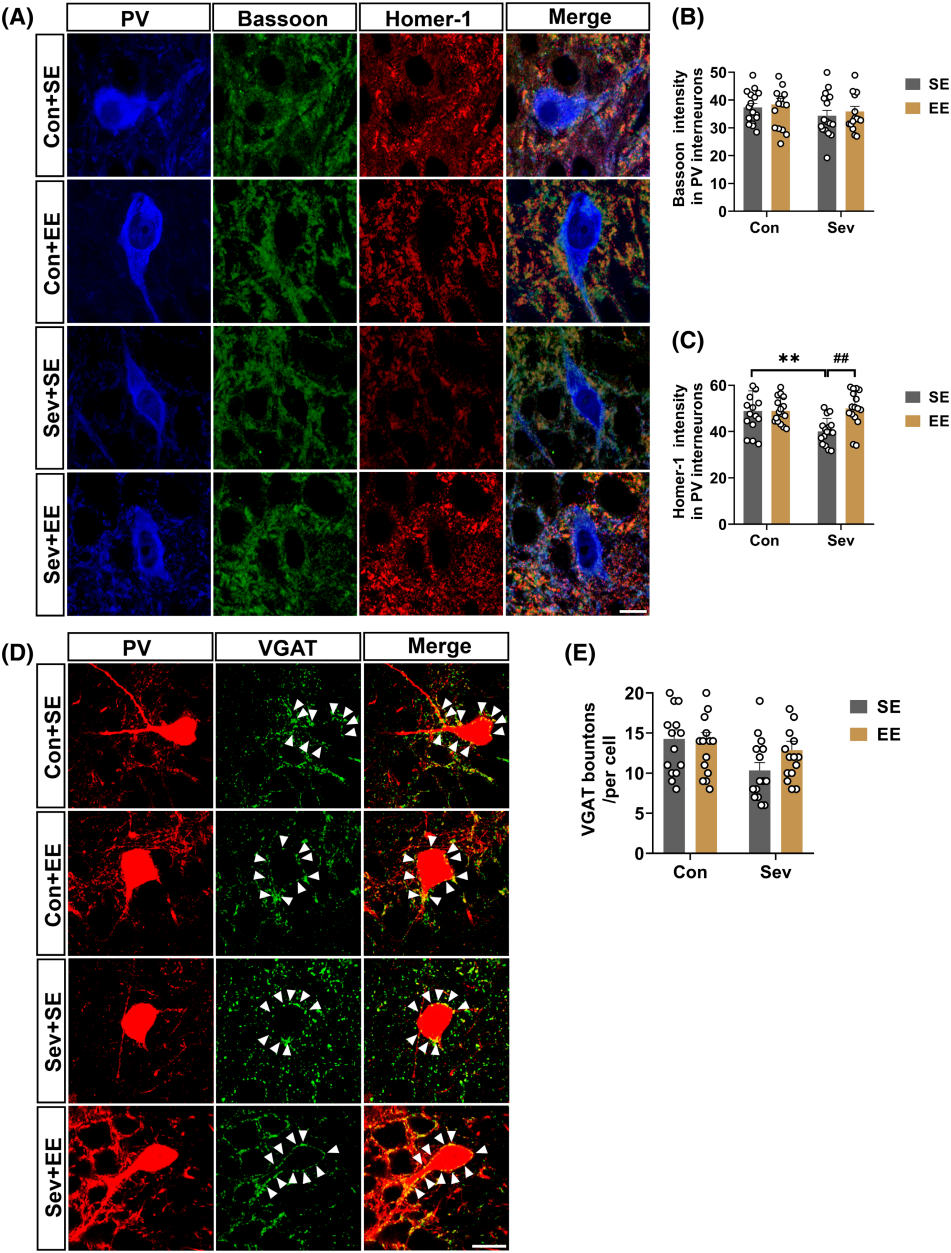
Enriched environments reversed sevoflurane‐induced decreased excitatory input onto PV interneurons. (A) Representative images of triple‐labeling immunostaining of PV (blue), bassoon (green), and homer‐1 (red) in the hippocampal CA3. Scale bar: 20 μm. (B, C) Quantitative bassoon (Interaction: *F*_(1,60)_ = 0.01460, *p* = 0.9042; Sev: *F*_(1,60)_ = 2.199, *p* = 0.1434; EE: *F*_(1,60)_ = 0.5107, *p* = 0.4776), and homer‐1 (Interaction: *F*_(1,60)_ = 7.771, *p* = 0.0071; Sev: *F*_(1,60)_ = 4.807, *p* = 0.0322; EE: *F*_(1,60)_ = 7.988, *p* = 0.0064) immunofluorescent intensity in PV interneurons in the hippocampal CA3 of control and sevoflurane‐exposed rats treated with SE or EE. (D) Representative images of double‐labeling immunostaining of PV (red) and vGat (green) in the hippocampal CA3. Scale bar: 20 μm. (E) Quantitative vGat in PV interneurons in the hippocampal CA3 of control and sevoflurane‐exposed rats treated with SE or EE (Interaction: *F*_(1,56)_ = 1.710, *p* = 0.1963; Sev: *F*_(1,56)_ = 5.204, *p* = 0.0264; EE: *F*_(1,56)_ = 1.007, *p* = 0.3198) Data are expressed as mean ± SEM (*n* = 3 rats/group). Results were analyzed by two‐way ANOVA followed by the Tukey post hoc test. **p < 0.01 vs. the Con + SE group. ##*p* < 0.01 vs. the Sev + SE group. Con, control; EE, enriched environment; PV parvalbumin; SE, standard environment; Sev, sevoflurane.

SynCAM1 exists in excitatory synapses in PV interneurons and regulates the excitability of PV.^23^ Triple‐labeling immunostaining for PV and presynaptic and postsynaptic markers of excitatory synapses (Bassoon and Homer‐1) and double‐labeling immunostaining PV and presynaptic markers of inhibitory synapses (vGat) was performed. Sevoflurane exposure resulted in a decreased immunofluorescent intensity of Homer‐1 in the PV interneurons, but exposure to EE significantly reversed sevoflurane‐induced decreased Homer‐1 immunoreactivity in PV interneurons (Interaction: *F*_(1,60)_ = 7.771, *p* = 0.0071; Sev: *F*_(1,60)_ = 4.807, *p* = 0.0322; EE: *F*_(1,60)_ = 7.988, *p* = 0.0064; Figure 7A,C). However, there were no significant differences observed in the immunofluorescent intensity of Bassoon and vGat in the PV interneurons in the hippocampal CA3 region among the four groups (bassoon: Interaction: *F*_(1,60)_ = 0.01460, *p* = 0.9042; Sev: *F*_(1,60)_ = 2.199, *p* = 0.1434; EE: *F*_(1,60)_ = 0.5107, *p* = 0.4776; vGat: Interaction: *F*_(1,56)_ = 1.710, *p* = 0.1963; Sev: *F*_(1,56)_ = 5.204, *p* = 0.0264; EE: *F*_(1,56)_ = 1.007, *p* = 0.3198; Figure 7A,B,D,E). Thus, these results suggest that EE specifically reversed the postsynaptic component of excitatory synapses in PV interneurons induced by sevoflurane exposure, likely through the restoration of synCAM1 expression.

### 3.7 SynCAM1 overexpression in PV interneurons in the hippocampal CA3 reversed the sevoflurane‐induced cognitive impairments

**FIGURE 8:**
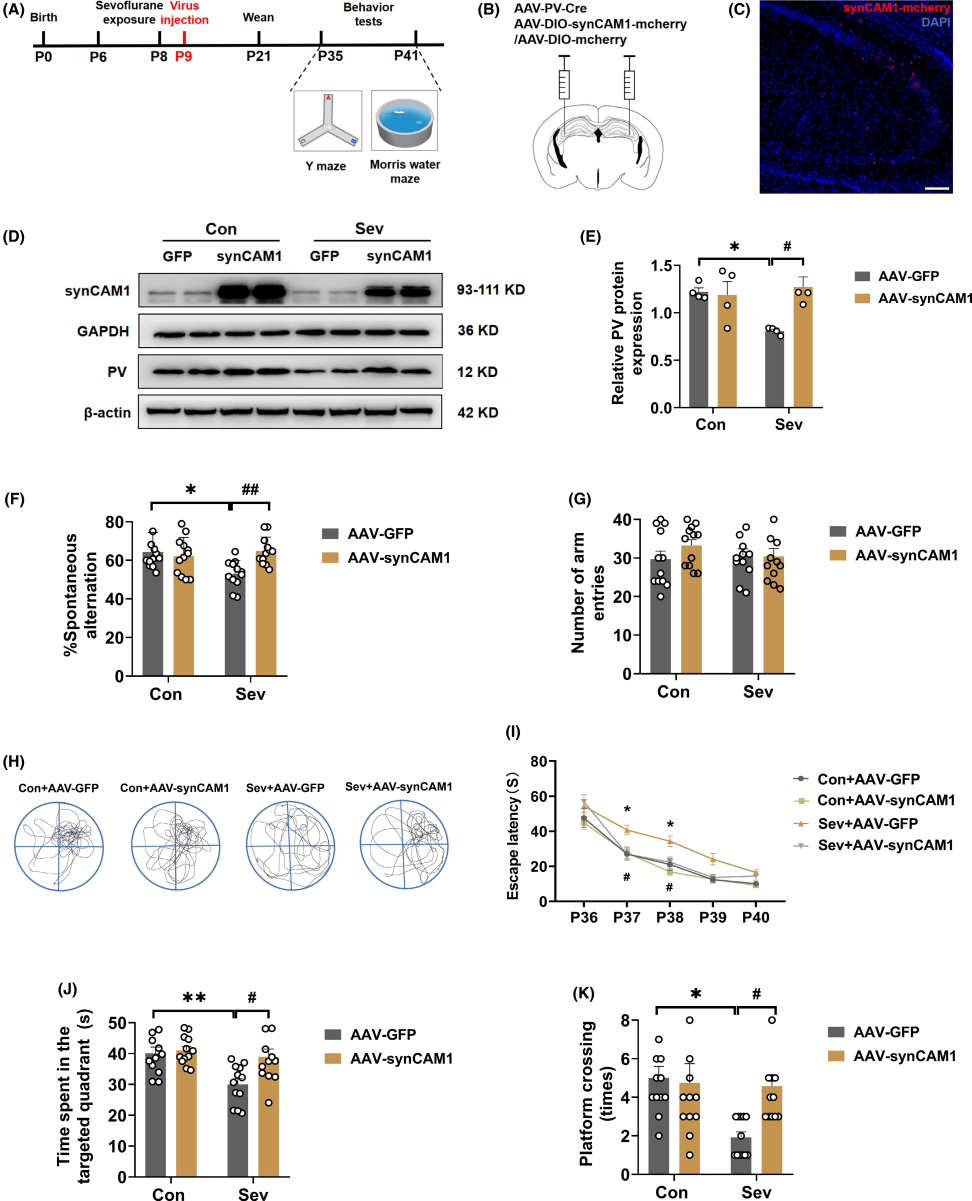
SynCAM1 overexpression in PV interneurons in the hippocampal CA3 reversed the sevoflurane‐induced decreased cognitive impairments. (A) Flowchart of synCAM1 overexpression and behavior tests. (B) Schematic showing virus injection of AAV‐PV‐Cre and AAV‐DIO‐synCAM1‐mCherry or AAV‐DIO‐mCherry into the hippocampal CA3. (C) Representative images showing the efficiency of synCAM1 overexpression on PV interneurons in hippocampal CA3. Scale bars: 200 μm. (D) Representative western blot bands of synCAM1 and PV in the hippocampus. (E) Quantitative analysis of PV protein level in control and sevoflurane‐exposed rats treated with AAV‐PV‐Cre, AAV‐GFP, or AAV‐synCAM1 (Interaction: *F*_(1,12)_ = 7.401, *p* = 0.0186; Sev: *F*_(1,12)_ = 3.189, *p* = 0.0994; SynCAM1 overexpression: *F*_(1,12)_ = 5.549, *p* = 0.0363). (F) Spontaneous alternation (Interaction: *F*_(1,44)_ = 7.952, *p* = 0.0072; Sev: *F*_(1,44)_ = 3.094, *p* = 0.0855; SynCAM1 overexpression: *F*_(1,44)_ = 3.830, *p* = 0.0567), and (G) number of arm entries of YMT (Interaction: *F*_(1,44)_ = 1.055, *p* = 0.3100; Sev: *F*_(1,44)_ = 0.2413, *p* = 0.6257; SynCAM1 overexpression: *F*_(1,44)_ = 0.7978, *p* = 0.3766) in control and sevoflurane‐exposed rats treated with AAV‐PV‐Cre and AAV‐DIO‐synCAM1‐mCherry or AAV‐DIO‐mCherry. (H) Representative swimming tracks of the control and sevoflurane‐exposed rats treated with AAV‐PV‐Cre and AAV‐DIO‐synCAM1‐mCherry or AAV‐DIO‐mCherry during the probe trial of the MWM test. (I) Escape latency (Interaction, *F*_(12,176)_ = 1.169, *p* = 0.3086; Time, *F*_(2.846,125.2)_ = 109.6, *p* < 0.0001; Treatment, *F*_(3,44)_ = 12.30, *p* < 0.0001), (J) spent time in the target quadrant (Interaction: *F*_(1,44)_ = 4.129, *p* = 0.0482; Sev: *F*_(1,44)_ = 9.583, *p* = 0.0034; SynCAM1 overexpression: *F*_(1,44)_ = 6.262, *p* = 0.0161), and (K) platform crossing times of the MWM test (Interaction: *F*_(1,44)_ = 4.797, *p* = 0.0339; Sev: *F*_(1,44)_ = 5.956, *p* = 0.0188; SynCAM1 overexpression: *F*_(1,44)_ = 3.293, *p* = 0.0764) in control and sevoflurane‐exposed rats treated with AAV‐PV‐Cre and AAV‐DIO‐synCAM1‐mCherry or AAV‐DIO‐mCherry. Data are expressed as mean ± SEM (*n* = 12 rats/group). The MWM test escape latency data were analyzed by two‐way repeated‐measures ANOVA followed by Tukey post hoc test, other results were analyzed by two‐way ANOVA followed by Tukey post hoc test. *p < 0.05, **p < 0.01 vs. the Con+AAV‐GFP group. #*p* < 0.05, ##*p* < 0.01 vs. the Sev + AAV‐GFP group. Con, control; MWM, Morris water maze; Sev, sevoflurane; YMT, Y maze test.

To further investigate the mechanism of synCAM1 reduction in the PV interneurons contributing to neonatal sevoflurane‐induced memory deficits, overexpression of synCAM1 in PV interneurons was achieved through stereotaxic hippocampal CA3 injections of AAV‐PV‐Cre and AAV‐DIO‐synCAM1‐mCherry or AAV‐DIO‐mCherry at P9. After 21 days, the YMT and MWM tests were conducted (Figure 8A,B). Immunofluorescence and western blot data confirmed the efficiency of virus transfection (Figure 8C,D). Furthermore, it was found that the overexpression of synCAM1 reversed the sevoflurane‐induced decreased PV protein level (Interaction: *F*_(1,12)_ = 7.401, *p* = 0.0186; Sev: *F*_(1,12)_ = 3.189, *p* = 0.0994; SynCAM1 overexpression: *F*_(1,12)_ = 5.549, *p* = 0.0363; Figure 8D,E). In the YMT, overexpression of synCAM1 blocked the decrease in spontaneous alternation observed in the sevoflurane‐exposed group (Interaction: *F*_(1,44)_ = 7.952, *p* = 0.0072; Sev: *F*_(1,44)_ = 3.094, *p* = 0.0855; SynCAM1 overexpression: *F*_(1,44)_ = 3.830, *p* = 0.0567; Figure 8F), and there were no differences in the total number of arm entries among the four groups (Interaction: *F*_(1,44)_ = 1.055, *p* = 0.3100; Sev: *F*_(1,44)_ = 0.2413, *p* = 0.6257; SynCAM1 overexpression: *F*_(1,44)_ = 0.7978, *p* = 0.3766; Figure 8G). In the MWM test, overexpression of synCAM1 also reversed the increase in the escape latency on P37 and P38 (Interaction: *F*_(12,176)_ = 1.169, *p* = 0.3086; Time: *F*_(2.846,125.2)_ = 109.6, *p* < 0.0001; Treatment: *F*_(3,44)_ = 12.30, *p* < 0.0001; Figure 8H,I), decrease in the target quadrant time (Interaction: *F*_(1,44)_ = 4.129, *p* = 0.0482; Sev: *F*_(1,44)_ = 9.583, *p* = 0.0034; SynCAM1 overexpression: *F*_(1,44)_ = 6.262, *p* = 0.0161; Figure 8J), and decrease in the platform crossing times induced by sevoflurane‐exposed (Interaction: *F*_(1,44)_ = 4.797, *p* = 0.0339; Sev: *F*_(1,44)_ = 5.956, *p* = 0.0188; SynCAM1 overexpression: *F*_(1,44)_ = 3.293, *p* = 0.0764; Figure 8K). These data indicate that overexpression of synCAM1 reversed spatial memory and working memory deficits by restoring the expression of PV.

### 3.8 SynCAM1 overexpression in PV interneurons in the hippocampal CA3 region reversed the sevoflurane‐induced the decreased gamma oscillations

**FIGURE 9:**
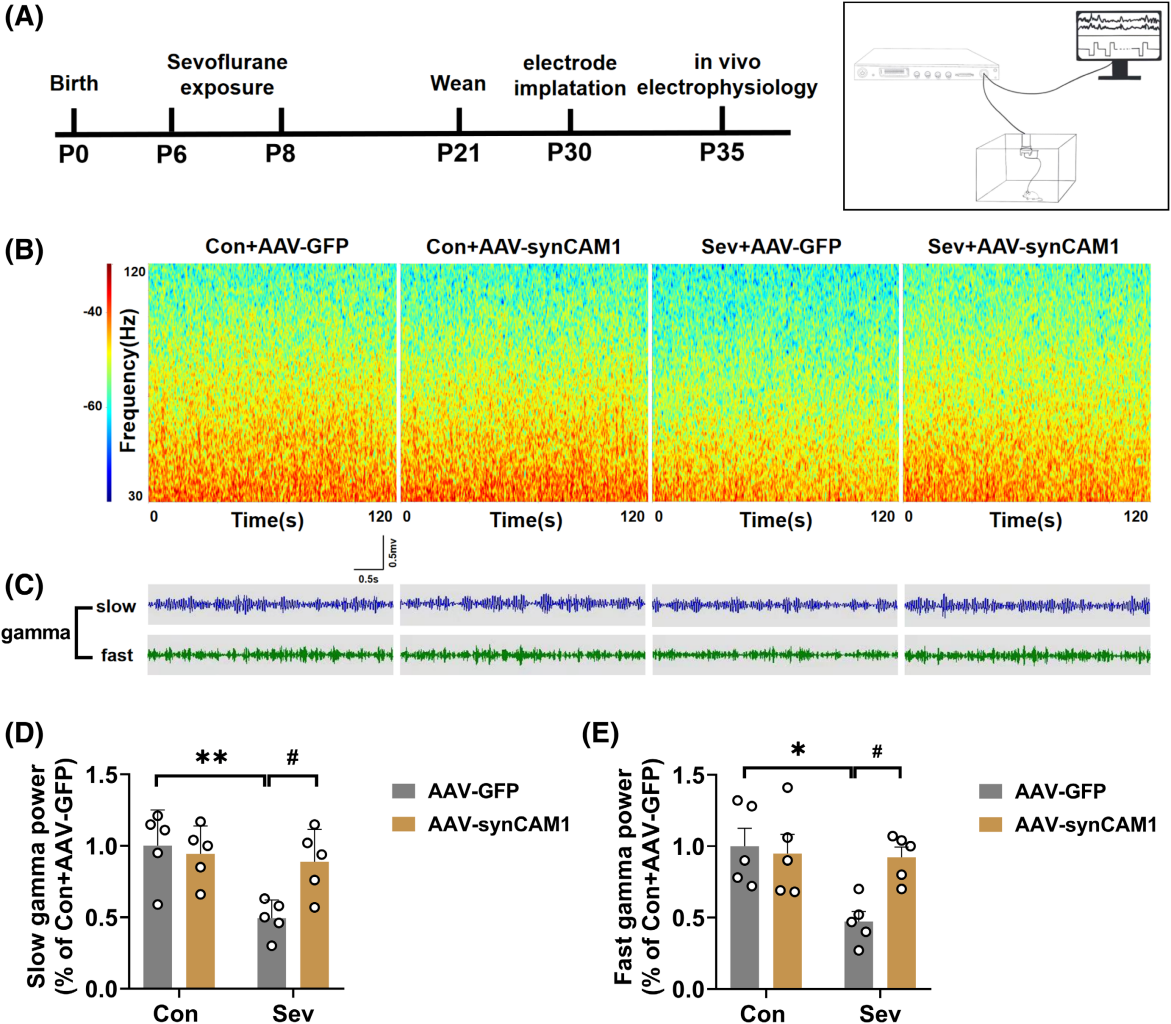
SynCAM1 overexpression in PV interneurons in the hippocampal CA3 region reversed the sevoflurane‐induced the decreased gamma oscillations. (A) Flowchart of synCAM1 overexpression and in vivo electrophysiology recording. (B) Representative gamma power spectra of local field potential hippocampal CA3. (C) Representative images of slow (30–50 Hz) and fast (65–120 Hz) gamma bands in the hippocampal CA3. (D, E) Quantification of slow (Interaction: *F*_(1,16)_ = 6.070, *p* = 0.0255; Sev: *F*_(1,16)_ = 9.450, *p* = 0.0073; SynCAM1 overexpression: *F*_(1,16)_ = 3.354, *p* = 0.0857) and fast (Interaction: *F*_(1,16)_ = 5.659, *p* = 0.0302; Sev: *F*_(1,16)_ = 6.892, *p* = 0.0184; SynCAM1 overexpression: *F*_(1,16)_ = 3.557, *p* = 0.0776) gamma power in the hippocampal CA3 in the control and sevoflurane‐exposed rats treated with AAV‐PV‐Cre and AAV‐DIO‐synCAM1‐mCherry or AAV‐DIO‐mCherry. Data are expressed as mean ± SEM (*n* = 5 rats/group). Results were analyzed by two‐way ANOVA followed by the Tukey post hoc test. *p < 0.05, **p < 0.01 vs. the Con+AAV‐GFP group. #*p* < 0.05 vs. the Sev + AAV‐GFP group. Con, control; Sev, sevoflurane.

A previous study showed that PV interneurons play a critical role in gamma oscillations (Kriener et al., 2022). Slow (30–50 Hz) and fast (65–120 Hz) gamma oscillations in the local field potential were recorded in the hippocampal CA3 in freely moving rats at P35 (Figure 9A). Sevoflurane exposure resulted in a decrease in both slow and fast gamma oscillations compared to those of the control group. However, overexpression of synCAM1 significantly blocked sevoflurane‐induced decreases in slow and fast gamma oscillations (slow: Interaction: *F*_(1,16)_ = 6.070, *p* = 0.0255; Sev: *F*_(1,16)_ = 9.450, *p* = 0.0073; SynCAM1 overexpression: *F*_(1,16)_ = 3.354, *p* = 0.0857; fast: Interaction: *F*_(1,16)_ = 5.659, *p* = 0.0302; Sev: *F*_(1,16)_ = 6.892, *p* = 0.0184; SynCAM1 overexpression: *F*_(1,16)_ = 3.557, *p* = 0.0776; Figure 9B–E).

### 3.9 SynCAM1 overexpression in PV interneurons in the hippocampal CA3 region reversed the sevoflurane‐induced dendritic spine loss

**FIGURE 10:**
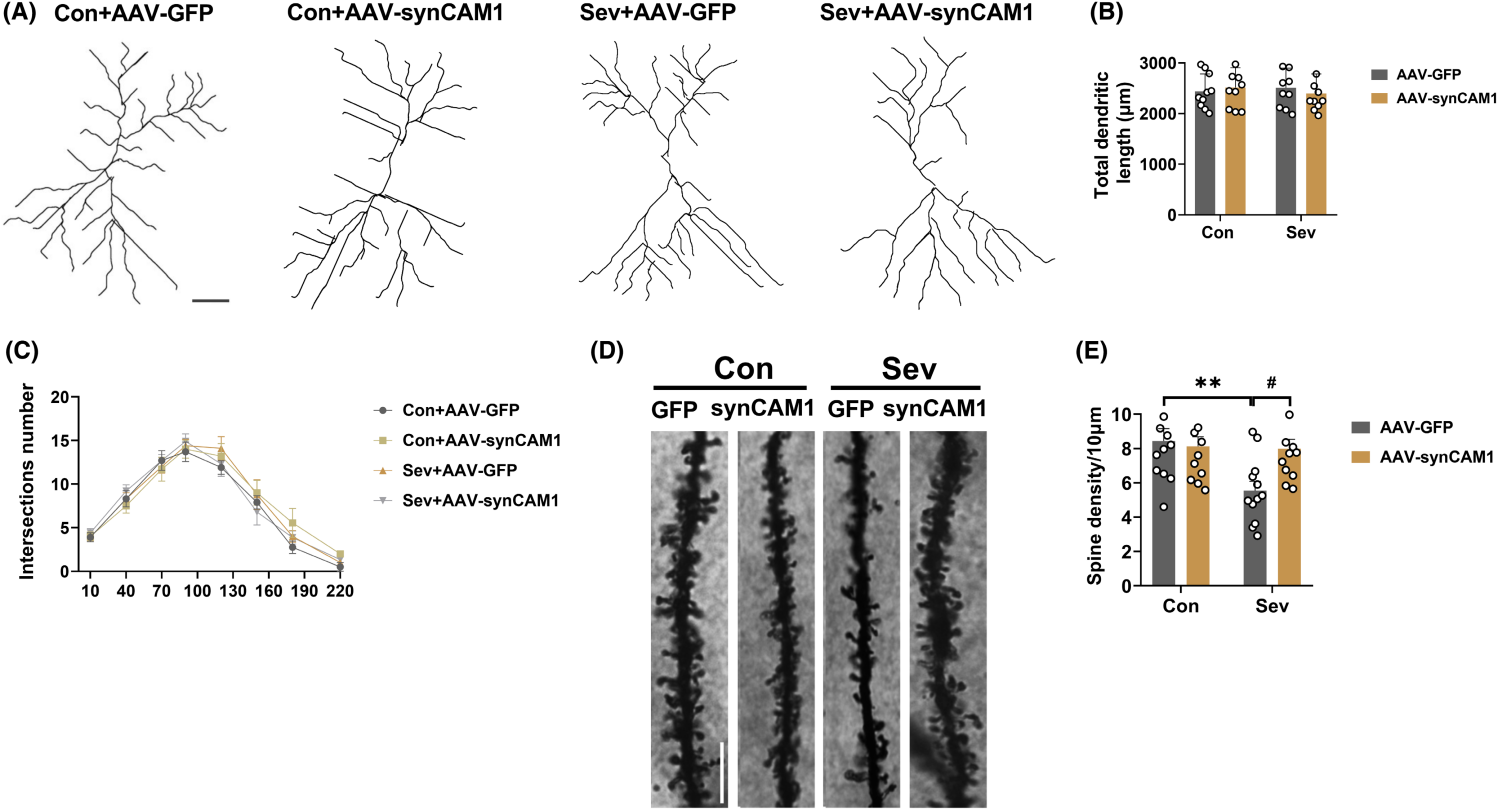
SynCAM1 overexpression in PV interneurons in the hippocampal CA3 region reversed the sevoflurane‐induced dendritic spine loss. (A) Representative images of tracings of morphological structures of hippocampal CA3 pyramidal neurons. Scale bars: 20 μm. (B) Quantification of total dendritic length (Interaction: *F*_(1,36)_ = 0.6509, *p* = 0.4251; Sev: *F*_(1,36)_ = 0.04155, *p* = 0.8396; SynCAM1 overexpression: *F*_(1,36)_ = 0.02805, *p* = 0.8679) and (C) intersection numbers of hippocampal CA3 pyramidal neurons in the control and sevoflurane‐exposed rats treated with AAV‐PV‐Cre and AAV‐DIO‐synCAM1‐mCherry or AAV‐DIO‐mCherry. (D) Representative images of dendritic spine density of hippocampal CA3 pyramidal neurons, Scale bars: 10 μm. (E) Quantification of the dendritic spine density of hippocampal CA3 pyramidal neurons in the control and sevoflurane‐exposed rats treated with AAV‐PV‐Cre and AAV‐DIO‐synCAM1‐mCherry or AAV‐DIO‐mCherry. (Interaction: *F*_(1,44)_ = 5.355, *p* = 0.0254; Sev: *F*_(1,44)_ = 6.454, *p* = 0.0147; SynCAM1 overexpression: *F*_(1,44)_ = 3.231, *p* = 0.0791). Data are expressed as mean ± SEM (*n* = 4 rats/group). Results were analyzed by two‐way ANOVA followed by the Tukey post hoc test. **p < 0.01 vs. the Con+AAV‐GFP group. #*p* < 0.05 vs. the Sev + AAV‐GFP group. Con, control; Sev, sevoflurane.

Additionally, overexpression of synCAM1 in PV interneurons was found to reverse sevoflurane‐induced dendritic spine loss examined using Golgi staining. No significant differences in the total dendritic length and intersection numbers were observed among the four groups (Interaction: *F*_(1,36)_ = 0.6509, *p* = 0.4251; Sev: *F*_(1,36)_ = 0.04155, *p* = 0.8396; SynCAM1 overexpression: *F*_(1,36)_ = 0.02805, *p* = 0.8679; Figure 10A–C). However, sevoflurane exposure resulted in a decrease of the dendritic spine density, while overexpression of synCAM1 significantly inhibited this loss induced by sevoflurane exposure in the hippocampal CA3 (Interaction: *F*_(1,44)_ = 5.355, *p* = 0.0254; Sev: *F*_(1,44)_ = 6.454, *p* = 0.0147; SynCAM1 overexpression: *F*_(1,44)_ = 3.231, *p* = 0.0791; Figure 10D,E). These results indicate that the sevoflurane‐induced reduction in synCAM1 expression in PV interneurons contributed to the impaired morphological integrity of pyramidal neurons, which was restored with synCAM1 overexpression.

## 4 DISCUSSION

In this study, we found that neonatal exposure to sevoflurane resulted in a decrease of synCAM1 expression in the PV interneurons, a reduction of PV interneurons phenotype, gamma oscillations decrease, and dendritic spine loss in the hippocampal CA3 region. Moreover, we uncovered that synCAM1 in the PV interneurons plays a role in the therapeutic effects of enriched environment (EE) treatment. We also demonstrated that overexpression of synCAM1 in the PV interneurons improved cognitive function by restoring PV expression, gamma oscillations, and dendritic spine density. Our findings suggest that dysregulation of synCAM1 in the PV interneurons contributes to cognitive impairments induced by neonatal sevoflurane exposure.

The brain during the growth spurt period is particularly susceptible to external stressors, including anesthesia agents.^29^ Preclinical studies showed that neonatal exposure to sevoflurane negatively affected brain development and behavior at adult age.^30^, ^31^ Consistent with these previous findings, our study revealed that neonatal exposure to sevoflurane led to spatial and working memory impairment assessed with the YMT and MWM test. Interestingly, deficits were not observed in the YMT,^5^ which may be attributed to differences in sevoflurane exposure methods, animal species, and behavior test times. The inhibitory network, comprising distinct classes of interneurons, is a crucial component of hippocampal microcircuits, controlling the spike timing of pyramidal neurons.^32^ Among these interneurons, PV interneurons have been identified as essential contributors to information processing and cognitive behavior, maintaining excitation‐inhibition homeostasis of neural circuits.^33^, ^34^ Furthermore, accumulating evidence linked the loss and dysfunction of PV interneurons to the pathophysiology of various neuropsychiatric disorders, such as schizophrenia, Alzheimer's disease, and postoperative cognitive impairments.^35^, ^36^, ^37^ PV interneurons begin to express at P6 and undergo final differentiation at P21, remaining stable at P30.^38^ During these critical developmental periods, PV interneurons have high metabolic demands and undergo activity‐dependent maturation, making them sensitive to stressors and drugs.^39^ However, the effect of sevoflurane exposure on PV expression is not yet fully understood. To shed light on the long‐term impact of sevoflurane exposure on PV interneuron expression, we tracked the developmental trajectories of PV expression after sevoflurane exposure. Our data revealed that neonatal sevoflurane exposure reduced the expression of PV interneurons at P14, P21, P28, and P35. Additionally, immunofluorescence data showed that sevoflurane exposure decreased the immunofluorescent intensity of GAD67 in the PV interneurons in the hippocampal CA3 region. Moreover, chemogenetic activation of PV interneurons in the hippocampal CA3 region reversed sevoflurane‐induced cognitive impairments, which is consistent with a previous study that found alterations of the PV neuronal activity in the hippocampus to regulate memory consolidation.^40^ These observations highlight the pivotal role of PV interneurons in memory deficits induced by sevoflurane exposure.

SynCAM1, specifically expressed in PV interneurons, plays a role in PV function.^22^, ^41^ During the developmental stage, synCAM1 protein is enriched in pre‐ and postsynaptic plasma membranes, linked to postsynaptic scaffold proteins, and is involved in synaptic plasticity.^18^, ^42^ Knockdown of synCAM1 selectively was reported to decrease its postsynaptic receptor in the PV interneurons, ultimately leading to PV dysfunction.^23^ Our study found that synCAM1 protein was detectable in the hippocampus as early as P7, with its expression increasing significantly through P14 and remaining high on P35, parallel to the developmental trajectories of PV interneurons.^38^ We also observed that neonatal sevoflurane exposure decreased the expression of synCAM1 at P14, P21, P28, and P35 in the hippocampus. Additionally, immunofluorescence data revealed that sevoflurane exposure decreased the immunofluorescence intensity of synCAM1 in the PV interneurons in the hippocampal CA3 region. These results suggest that synCAM1 may undergo cell‐type and region‐specific changes in the hippocampus.

During critical brain developmental periods, rats raised in an enriched environment (EE) cage receive enhanced social interaction and sensory and motor stimulation, which can be rewarding and motivational.^43^ Several studies have identified that EE improves cognitive impairment in various animal models of neurological and psychiatric disorders, including sevoflurane‐induced cognitive impairments.^44^ EE may improve brain function by promoting neurogenesis and synapse function.^45^ Our data demonstrated that EE treatment ameliorated the cognitive impairment induced by sevoflurane exposure. Moreover, EE treatment reversed the expression of PV interneurons and synCAM1 in PV interneurons in the sevoflurane exposure group. A recent animal study also showed that EE prevents neonatal sevoflurane‐induced PV interneuron developmental delays in the hippocampus.^38^ Additionally, we found that neonatal sevoflurane exposure reduced excitatory input to PV interneurons by affecting the postsynaptic component change of excitatory synapses in PV interneurons, which was rescued by EE treatment. Previous findings demonstrated that EE‐induced hippocampal neural progenitor proliferation and neurogenesis contribute to the number and function of PV interneurons.^46^ EE also promotes hippocampal PV network configurations to enhance synaptic plasticity.^47^ Our study suggests that PV interneurons and synCAM1 in PV interneurons may play a mechanistic role in the alleviation of sevoflurane‐induced cognitive impairment through EE treatment.

To explore the mechanism of synCAM1 reduction in PV interneurons in neonatal sevoflurane‐induced memory deficits, we employed a viral strategy to overexpress synCAM1 in PV interneurons. Remarkably, the overexpression of synCAM1 in PV interneurons rescued the sevoflurane‐induced decrease in PV expression and cognitive impairment. These data strongly suggest that synCAM1 is involved in sevoflurane‐induced cognitive dysfunction by affecting PV expression. Notably, PV interneurons play a crucial role in synchronizing the firing of excitatory pyramidal cells through fast synaptic inhibition, which is essential for generating and maintaining gamma oscillations in the brain.^48^ Extensive evidence has shown that gamma oscillations (30–120 Hz) are a prominent feature in many brain areas and support higher cognitive processes, such as working memory.^49^ Gamma oscillations can be further divided into slow and fast gamma oscillations, mediating separate information transmission.^50^ Slow gamma oscillations are generated in CA3 and are necessary for memory retrieval, while fast gamma oscillations propagate from the entorhinal cortex, playing a role in new memory encoding.^51^ Abnormal gamma oscillations have been implicated in the pathogenesis of schizophrenia, Alzheimer's disease, and other mental and neurodegenerative disorders.^52^, ^53^ SynCAM1 is crucial for maintaining neural network connectivity by controlling the development of excitatory synaptic contacts^22^, ^54^ and improving excitatory inputs onto PV interneurons to regulate the synchronization of CA3 network activity.^23^ However, whether gamma oscillations are involved in the mechanism of general anesthetic agents has not been reported yet. To shed light on the neural mechanisms underlying sevoflurane‐induced cognitive deficits, we assessed oscillatory activities in the hippocampal CA3 region of rats on P35. Our findings showed that neonatal exposure to sevoflurane led to impaired average slow and fast gamma power in the hippocampal CA3. Interestingly, overexpression of synCAM1 rescued the decreased gamma oscillations induced by sevoflurane exposure. Given that synCAM1 specifically expresses in PV interneurons and regulates their excitability, we speculate that sevoflurane‐induced synCAM1 loss affects the expression of PV interneurons, subsequently impairing gamma oscillations.

Furthermore, sevoflurane‐induced neurotoxicity and long‐term cognitive impairment have been linked to the impaired structure of pyramidal neurons.^55^ Our results revealed that sevoflurane‐exposed rats exhibited a lower density of dendritic spines, which was reversed by restoring the expression of synCAM1 in PV interneurons. Despite PV interneurons accounting for only a small fraction of all neurons in the hippocampus, changes in their activity can have amplified effects through the consequent activation of excitatory neurons.^56^ Previous research has shown that selective activation of PV interneurons prevents dendritic spines loss.^57^ The hypo‐inhibition of PV interneurons contributes to the hyperactivity of pyramidal neurons, potentially leading to the loss of dendritic spines through a homeostatic mechanism.^58^ These findings suggest that synCAM1 signaling in PV interneurons plays a crucial role in maintaining the morphological integrity of pyramidal neurons in the hippocampal CA3 region.

In conclusion, our study highlights the importance of hippocampal synCAM1 in PV interneurons in neonatal sevoflurane‐induced cognitive impairments. Enriched environment (EE) treatment rescues cognitive impairment by restoring the expression of PV interneurons and synCAM1 in PV interneurons. Furthermore, overexpressing synCAM1 can also rescue cognitive impairment by restoring the expression of PV interneurons, gamma oscillations, and dendritic spine density. These findings suggest that targeting synCAM1 in the hippocampus may hold promise as a potential treatment for sevoflurane‐induced cognitive impairments.

## AUTHOR CONTRIBUTIONS

J.J.Y. and J.M. designed research; M.M.Z., H.W.G., and T.T.Z. performed all experiments; M.M.Z., P.M.L., and W.T.P analyzed the data; M.M.Z., J.M., and H.W.G. wrote the manuscript; J.J.Y. and J.M. supervised the overall experiment. All authors have approved the final version of the manuscript.

## CONFLICT OF INTEREST STATEMENT

The authors have no conflicts of interest to declare.

## Supporting information


Figure S1

## Data Availability

Data supporting the findings of this study are available within the article.
