# Supplementary material for: SynCAM1 deficiency in the hippocampal parvalbumin interneurons contributes to sevoflurane‐induced cognitive impairment in neonatal rats
Source: CNS Neurosci Ther. 2023 Dec 17;30(1):e14554. doi: 10.1111/cns.14554 (PMC10805405; doi:10.1111/cns.14554)
Supplement: Supplementary file 1 — Figure S1 [file CNS-30-e14554-s001.zip › cns14554-Legend.docx]

**Supplemental Figure 1. Sevoflurane does not change synCAM1 expression in SST and VIP interneurons in the hippocampus on P35**

1. Representative images of double-labeling immunostaining of SST (red) and synCAM1 (green) in the hippocampus. Scale bar: 50 µm (overview), 20 µm (zoom). **(B)** Quantitative synCAM1 immunofluorescent intensity in SST interneurons in the hippocampal CA1 (t_(28)_ =1.588, P = 0.8750), CA3 (t_(28)_ = 0.7757, P = 0.4444), DG (t_(28)_ = 0.1.225, P = 0.2308) of the control and sevoflurane-exposed rats on P35. **(C)** Representative images of double-labeling immunostaining of VIP (red) and synCAM1 (green) in the hippocampus. Scale bar: 50 µm (overview), 20 µm (zoom). **(D)** Quantitative synCAM1 immunofluorescent intensity in VIP interneurons in the hippocampal CA1 (t_(28)_ = 0.1121, P = 0.9115), CA3 (t_(28)_ = 0.6293, P = 0.5343), DG (t_(28)_ = 1.217, P = 0.2337) of control and sevoflurane-exposed rats on P35. Data are expressed as mean ± SEM (n = 3 rats/group). Results were analyzed by unpaired student’s t-test. Con, control; Sev, sevoflurane.
