# Supplementary figures and images for: SynCAM1 deficiency in the hippocampal parvalbumin interneurons contributes to sevoflurane‐induced cognitive impairment in neonatal rats
Source: CNS Neurosci Ther. 2023 Dec 17;30(1):e14554. doi: 10.1111/cns.14554 (PMC10805405; doi:10.1111/cns.14554)

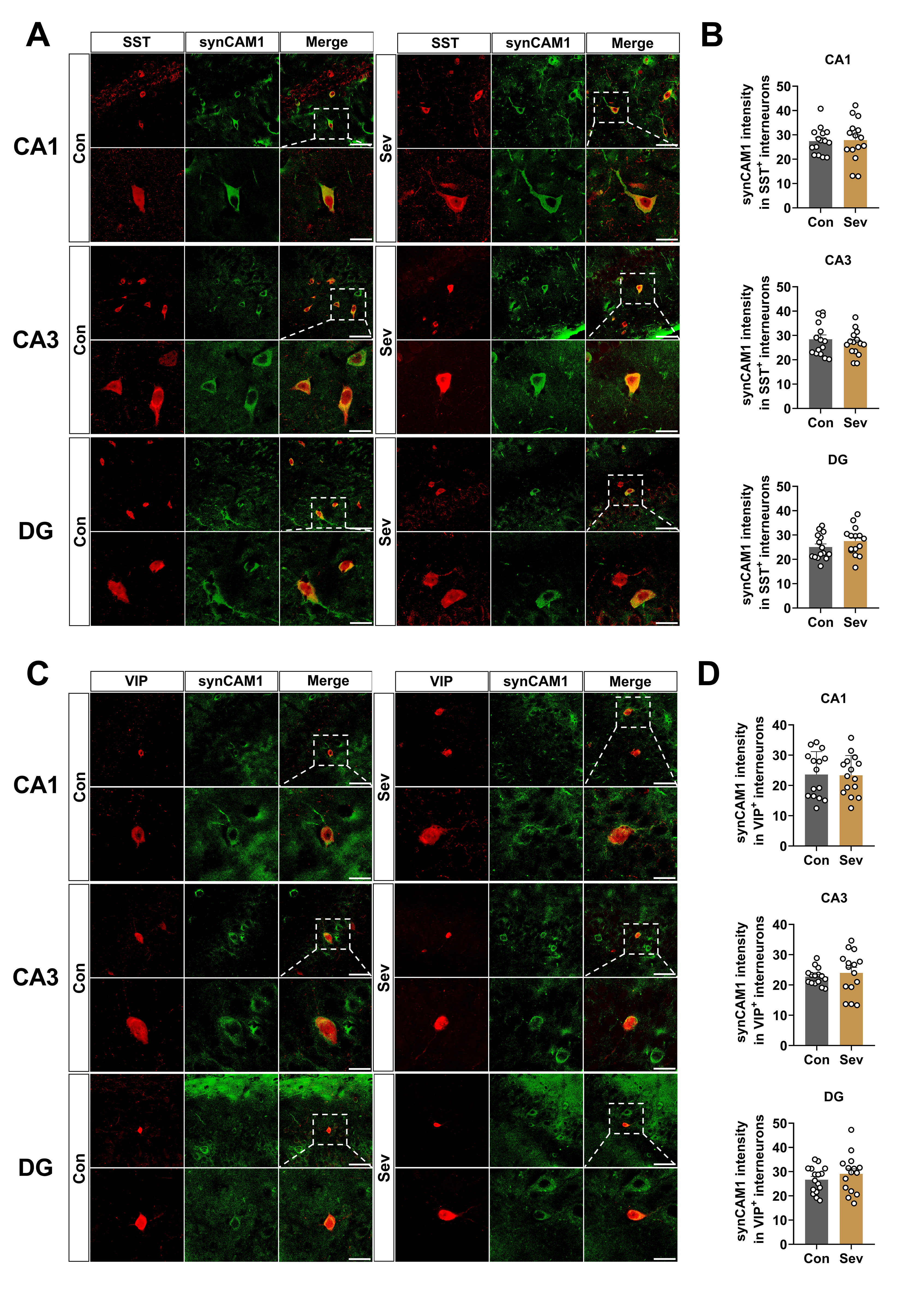

Supplement: Supplementary file 1 — Figure S1 [file CNS-30-e14554-s001.zip › cns14554-sup-0001-FigureS1.tif]
